# Broadband Complex Permittivity Spectra: Cole–Cole vs Circuit Models

**DOI:** 10.1021/acsmeasuresciau.5c00065

**Published:** 2025-09-22

**Authors:** Farizal Hakiki, Chih-Ping Lin

**Affiliations:** † Disaster Prevention & Water Environment Research Center (DPWE), 34914 National Yang Ming Chiao Tung University, Hsinchu 300, Taiwan; ‡ Civil Engineering Department, 34914 National Yang Ming Chiao Tung University, Hsinchu 300, Taiwan

**Keywords:** complex permittivity, impedance spectroscopy, time-domain reflectometry, Cole−Cole, Pelton

## Abstract

Hydraulic properties such as porosity, water, and clay content can be inferred from electrical parameters like permittivity, conductivity, and resistivity. Spectral data enhance this analysis by revealing features such as pore size and clay type in wet particulate media. In liquid samples, electrode polarization is clearly observed, as orientational polarization occurs only at higher frequencies (MHz to sub-GHz). In contrast, particulate media exhibit electrode polarization artifacts that obscure spatial polarization peaks within the Hz–MHz range, especially in highly conductive materials like wet clayey soils, making the Cole–Cole model insufficient for distinguishing these effects. Therefore, a general circuit model using a parallel form of a resistor and a constant phase element configuration more effectively separates inherent material polarization from electrode polarization. The electrode polarization limiting frequency (*f*_EP_) correlates with both material conductivity and electrode properties, even with low-polarization electrodes like Ag/AgCl. A novel method is introduced to estimate the effective constant phase element exponent ( $\tilde{\eta }$ ) using the slope of log permittivity vs log frequency. Finally, the chargeability of kaolinite (*m* = 0.83–0.86), derived from the ratio of critical frequencies between the Cole–Cole and Pelton models, aligns with its fundamental definition: *m* = (σ_∞_ – σ_0_)/σ_∞_, where σ_0_ is the DC conductivity and σ_∞_ is the high-frequency conductivity.

## Graphical abstract

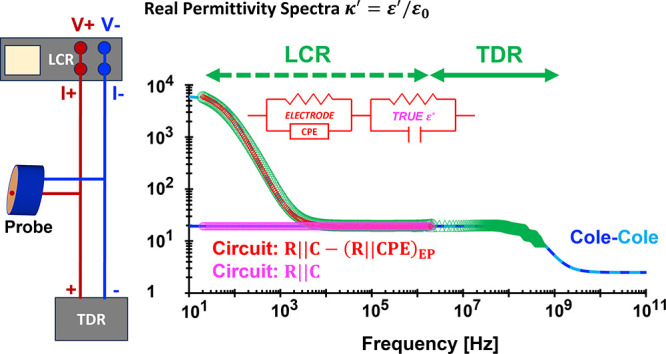

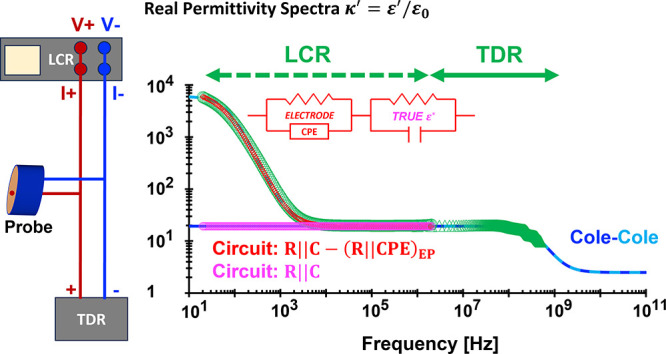

## 1 Introduction

We can estimate hydraulic properties of geo-materials, such as porosity, water content, and clay content by examining their electrical properties, including permittivity, conductivity, and resistivity.^1^ For example, permittivity, which reflects a material’s ability to become polarized due to an electric field, can provide insights into the amounts of pore fluid,^2,3^ type of pore fluids,^4,5^ porosity,^6,7^ and specific surface area.^8–10^ Similarly, conductivity and its inverse, resistivity, which measure a material’s ability to allow the flow of electric current, can provide information about the salinity of pore fluid,^11,12^ porosity,^8,13^ multiphase saturations,^5,14^ and clay content.^3,15^

Researchers can gain deeper insights into these hydraulic properties by analyzing spectral data across a range of frequencies.^1^ Spectral analysis enables the identification of finer details, such as pore throat,^8,16,17^ pore size distribution,^18,19^ permeability,^16,17,20^ specific surface area,^15,17^ and the type of clays.^15,21^ The type of clay is particularly substantial, as it relates to the material’s specific surface area, which influences its ability to retain water and ions.^22,23^ In wet particulate media, where water and solid particles coexist, these spectral techniques are indispensable for understanding the interactions between these two phases.^1,24–27^

One of the measurement artifacts in permittivity spectra obtained through impedance spectroscopy is electrode polarization, which is typically observed below 1 kHz.^11,26,28–32^ In the case of liquid samples, this effect is relatively straightforward to detect because orientational polarization, i.e., the alignment of dipoles within the material occurs only at higher frequencies, typically in the MHz to sub-GHz range.^11,33,34^ However, when dealing with particulate and porous media, the situation becomes more complex.^26,31,32^ Electrode polarization artifacts often interfere with the detection of spatial polarization peaks within a material’s complex permittivity spectrum. These artifacts are particularly problematic in the Hz to MHz frequency range, where details about spatial polarizations are often obscured.^26,31,35^ This interference represents a major challenge in distinguishing and interpreting spatial polarization effects.^1^

The challenge becomes even more pronounced in highly conductive materials, such as wet soils with a high clay content or high specific surface area,^21,36^ higher degree of saturation,^21,26^ or increased pore fluid salinity.^4,36^ The increased conductivity in these materials, driven by the abundance of free ions and counterions of clay, exacerbates the problem because the electrode polarization is considerably amplified.^26^ Previous studies suggest that spatial polarizations are better seen in materials with low water content or pore-fluid conductivity.^37^

Traditional models, such as the Cole–Cole model often used to interpret permittivity spectra, are inadequate to account for the complexities introduced by strong electrode polarization. As a result, the role of spatial polarizability in permittivity spectra remains unresolved, leaving a critical gap in understanding the material’s electrical behavior.^1^

To address these challenges, this study aims to undertake the following actions and objectives:

1. Employ advanced circuit models to simulate the underlying physical processes of governing polarizations. These models are designed to elucidate the intricate interactions between electrode polarization and spatial polarization effects.
2. Clarify the diverse terminology used across disciplines. Material scientists and chemists typically focus on complex permittivity, often using the Cole–Cole model to characterize dielectric properties.^11,38^ In contrast, geoscientists commonly use terms like complex conductivity or resistivity, applying frameworks such as Cole–Cole and Pelton’s models to describe subsurface electrical properties.^39,40^ Pelton’s model is essentially a circuit-based model expressed in resistivity form rather than impedance. These disciplinary preferences underscore the need for a unified approach to interpreting electrical data. Therefore, comprehensive comparisons between the Cole–Cole model and circuit models are necessary.

## 2 Revisited Theory

### 2.1 Conductivity vs Permittivity

Ohm’s law states: ***J*** = σ_eff_^*^ ***E***, where ***J*** is the current density, σ_eff_^*^ is the effective complex conductivity, and ***E*** is the applied electric field (Figure 1). This field induces a voltage drop Δ*V* over sample length *L*, given by |Δ*V*| = | ***E*** |*L*. The subscript “eff” denotes the material’s overall effective properties. We express current density magnitude *J* from the current *I* that passes through a cross-sectional area *A* such that *J* = *I*/*A*. Thus, Kirchoff’s reformulation of Ohm’s law in circuit form is *I*/*A* = σ_eff_^*^|Δ*V*|/*L*. The complex impedance Z* = Δ*V*/*I* relates to conductivity via a geometric factor β = *A*/*L*:^1,11,41^

$$\sigma_{\mathrm{eff}}^{*}=\frac{1}{\beta Z^{*}}$$ 1

**Figure 1:**
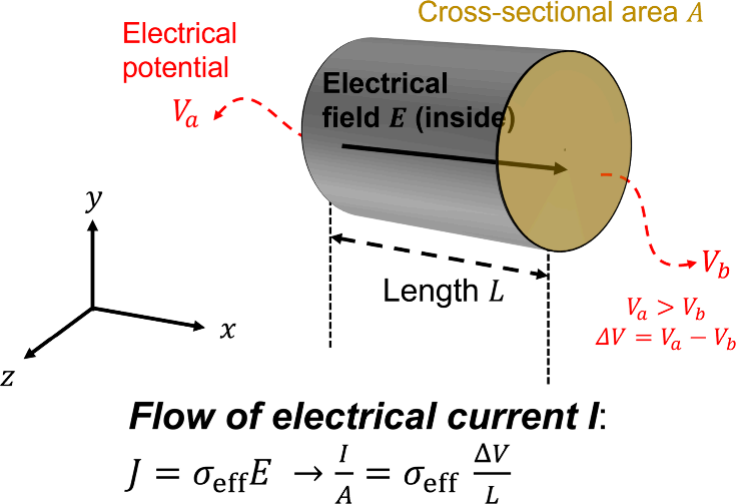
Illustration of Ohm’s law applied to a cut-section of a cylindrical container (or cable). All variables are represented in scalar form unless indicated in bold. The electric field **E** is oriented in the *x*-direction, and the cross-sectional area *A* is in the *y*–*z* plane with the associated normal vector **n̂** also aligned along the *x*-direction. Electrical potentials *V*_a_ and *V*_b_ are measured on surfaces lying in the *y*–*z* plane.

The reciprocal form of conductivity is resistivity, such that ρ_eff_^*^ = 1/σ_eff_^*^.

The total current density (***J*** = σ_eff_^*^ ***E***) combines DC conduction (***J***_**C**_ = σ_DC_ ***E***) and displacement current density (***J***_**D**_ = *∂* ***D***/*∂t*), such that ***J*** = ***J***_**C**_ + ***J***_**D**_. The displacement field ***D*** = ε* ***E*** represents the polarization response in a material with permittivity ε* ^39,40,42,43^ and leads to ***J***_**D**_ = *j*ωε* ***E*** for a time-harmonic field ***E = E***_**0**_ *e* ^*j*ω*t*^. Thus, the total current density is *J* = (σ_DC_ + *j*ωε*) ***E*** and the subsequent effective complex conductivity is σ_eff_^*^ = σ_DC_ + *j*ωε*; where $j=\sqrt{-1}$ and ω is angular frequency, 2π*f*.

We can eventually establish the relationship between effective complex conductivity (σ_eff_^*^ = σ_eff_^′^ + *jσ*_eff_^″^) and permittivity (ε* = ε′ – *j*ε″):^1^

$$\sigma_{\mathrm{eff}}^{*}=j\omega \varepsilon_{\mathrm{eff}}^{*}$$ 2

$$\varepsilon_{\mathrm{eff}}^{*}=-j\frac{\sigma_{\mathrm{DC}}}{\omega }+\varepsilon^{*}=\varepsilon^{\prime }-j\left(\varepsilon^{\prime \prime }+\frac{\sigma_{\mathrm{DC}}}{\omega }\right)$$ 3

$$\{\begin{array}{c} \sigma_{\mathrm{eff}}^{\prime }=\sigma_{\mathrm{DC}}+\omega \varepsilon^{\prime \prime }\\ \sigma_{\mathrm{eff}}^{\prime \prime }=\sigma^{\prime \prime }=\omega \varepsilon^{\prime } \end{array}$$ 4

The effective imaginary permittivity is given by $\varepsilon_{\mathrm{eff}}^{\prime \prime }=\varepsilon^{\prime \prime }+\frac{\sigma_{\mathrm{DC}}}{\omega }$ , capturing both polarization ε″ at high frequencies and DC conduction σ_DC_ at low frequencies. Thus, the effective complex permittivity can be expressed as ε_eff_^*^ = ε′ – *j*ε_eff_^″^ and ε′ = ε_eff_^′^. In relative form with respect to the vacuum permittivity ε_0_ = 8.854187 × 10^–12^ F/m, the effective complex permittivity is expressed as ε_eff_^*^ = κ_eff_^*^ε_0_, consequently, κ_eff_^*^ = $\kappa_{\mathrm{eff}}^{\prime }-j\kappa_{\mathrm{eff}}^{\prime \prime }=\kappa^{\prime }-j(\kappa^{\prime \prime }+\frac{\sigma_{\mathrm{DC}}}{\omega \varepsilon_{0}})$ where $\kappa_{\mathrm{eff}}^{\prime }=\kappa^{\prime }$ .

### 2.2 Impedance vs Permittivity

Low-frequency methods such as impedance spectroscopy or spectral induced polarization yield the impedance magnitude |Z*| and impedance phase angle θ_*Z*_. The spectral plot of |Z*| and θ_*Z*_ is called a Bode plot. Complex impedance is defined as Z* = *Z*′ + *jZ*″ = |Z*| cos θ_*Z*_ + *j*|Z*| sin θ_*Z*_. These methods provide typical phase angles (−2π ≤ θ_*Z*_ ≤ 0) and sinθ_*Z*_ ≤ 0, therefore, −*Z*″ ≥ 0. That is why a Nyquist plot (−*Z*″ vs *Z*′) typically lies in the first quadrant: *Z*′ represents resistance *R* (definite positive), while *Z*″ designates reactance *X*, typically negative.

From eqs 1 and 2, the effective complex permittivity (ε_eff_^*^= ε′ – *j*ε_eff_^″^) becomes^11,41^

$$\varepsilon_{\mathrm{eff}}^{*}=\frac{1}{j\beta \omega Z^{*}}$$ 5

$$\{\begin{array}{l} \varepsilon^{\prime }=\frac{-1}{\beta \omega }\frac{Z^{\prime \prime }}{|Z^{*}{|}^{2}}=\frac{-1}{\beta \omega }\frac{\sin\;\theta_{Z}}{\left|Z^{*}\right|}\\ \varepsilon_{\mathrm{eff}}^{\prime \prime }=\frac{1}{\beta \omega }\frac{Z^{\prime }}{|Z^{*}{|}^{2}}=\frac{1}{\beta \omega }\frac{\cos\;\theta_{Z}}{\left|Z^{*}\right|} \end{array}$$ 6

These relations show that complex permittivity can be determined independently of any assumed circuit model, using only the measured frequency-dependent impedance magnitude |Z*|, phase angle θ_*Z*_, and the geometric factor β.

### 2.3 Cole–Cole Model

Debye model is the oldest equation that fits complex permittivity spectra:^44^

$$\varepsilon^{*}=\varepsilon_{\infty }+\frac{\varepsilon_{s}-\varepsilon_{\infty }}{1+j\omega \tau }$$ 7

The measured ε″ data often exhibits a certain spread at the frequency around the Debye peak observed in the plot of ε″ vs log ω. The Cole–Cole model accommodates this broadening effect:^45^

$$\varepsilon^{*}=\varepsilon_{\infty }+\frac{\varepsilon_{s}-\varepsilon_{\infty }}{1+(j\omega \tau {)}^{\delta }}$$ 8

Here, ε_s_ and ε_∞_ represent the static (DC) and high-frequency permittivity, respectively. When expressed as relative permittivity κ* = ε*/ε_0_, the corresponding limits are κ_s_ and κ_∞_. As ω → 0, ε* → ε_s_; as ω → ∞, ε* → ε_∞_ since (ε_s_ – ε_∞_) ≪ 1 + (*j*ωτ)^δ^. The broadening factor δ controls the distribution of an enormous number of molecular relaxations, seen in the ε″ at the Debye peak or critical relaxation frequency *f*_c_ = (2πτ)^−1^ and 0 ≤ δ ≤ 1.

The Debye model offers a simpler decomposition of complex permittivity ε* = ε′ – *j*ε″ than the Cole–Cole model. Its analytical form is

$$\varepsilon^{*}=\varepsilon_{\infty }+\frac{\varepsilon_{s}-\varepsilon_{\infty }}{1+j\omega \tau }=\{\begin{array}{l} \varepsilon^{\prime }=\varepsilon_{\infty }+\frac{\left(\varepsilon_{s}-\varepsilon_{\infty }\right)}{1+\omega^{2}\tau^{2}}\\ \varepsilon^{\prime \prime }=\frac{(\varepsilon_{s}-\varepsilon_{\infty })\omega \tau }{1+\omega^{2}\tau^{2}} \end{array}$$ 9

To account for DC conduction, the effective imaginary permittivity becomes $\varepsilon_{\mathrm{eff}}^{\prime \prime }=\frac{\sigma_{\mathrm{DC}}}{\omega }+\varepsilon^{\prime \prime }$ , leading to an extended Cole–Cole model:^1,11^

$$\varepsilon_{\mathrm{eff}}^{*}=-j\frac{\sigma_{\mathrm{DC}}}{\omega }+\varepsilon_{\infty }+\frac{\varepsilon_{s}-\varepsilon_{\infty }}{1+(j\omega \tau {)}^{\delta }}$$ 10

The decomposition of eq 10 into its real (ε′) and effective imaginary (ε_eff_^″^) components is presented in the Supporting Information. For materials with multiple relaxations, such as porous media, the model extends to^1,11^

$$\varepsilon_{\mathrm{eff}}^{*}=-j\frac{\sigma_{\mathrm{DC}}}{\omega }+\sum_{i}^{N}\left[\varepsilon_{L_{i}}+\frac{\varepsilon_{U_{i}}-\varepsilon_{L_{i}}}{1+(j\omega \tau_{i}{)}^{\delta_{i}}}\right]$$ 11

Here, each polarization process is bounded by its upper (ε_*U*_) and lower (ε_*L*_) permittivity limits.

### 2.4 Circuit Elements

Field-scale electrical property measurements often use wave propagation methods, e.g., ground-penetrating radar or electromagnetic induction. To apply Maxwell’s equations and transmission line theory, the wavelength λ must span multiple cycles within the test length *L*, satisfying λ/*L* ≪ 1. At large scales (10 m to kilometers), this remains valid with long wavelengths or low-frequency waves, as in airborne electromagnetic surveys. The same condition applies to high-frequency lab measurements where the small *L* ranges from 1 mm to 10 cm.^1^

In contrast, low-frequency lab measurements involve small samples where λ/*L* ≫ 1, making wave-based analysis invalid.^1^ In this regime, lumped-circuit models are appropriate. The measured impedance is represented using elements like resistors *R*, capacitors *C*, inductors $\tilde{L}$ , or constant phase elements (CPE). Table 1 details the impedance expressions for each circuit component.

**Table 1:**
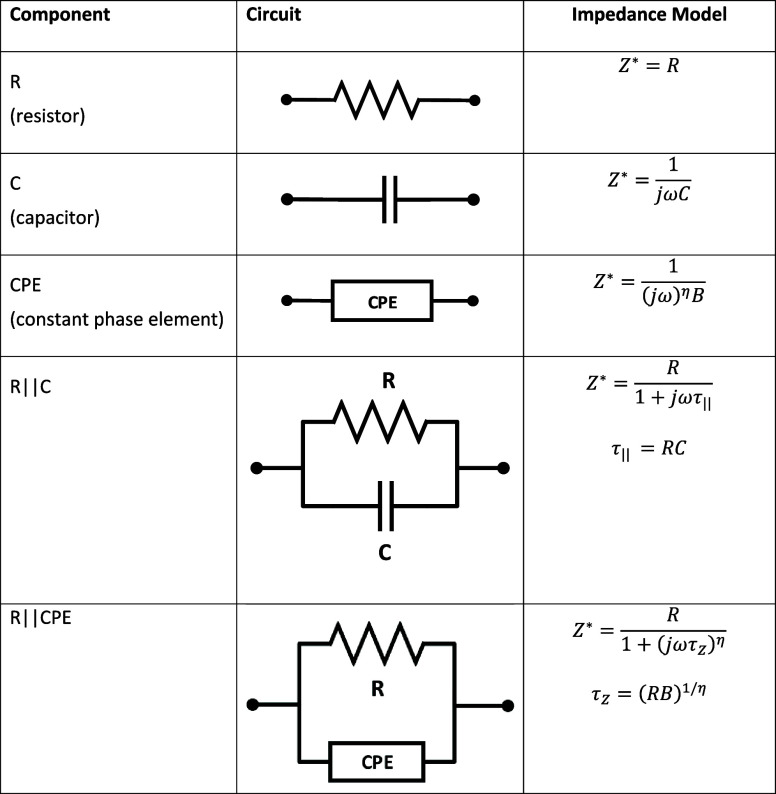
Circuit Component and Impedance

The CPE impedance is $Z_{\mathrm{CPE}}=\frac{1}{(j\omega {)}^{\eta }B}$ , when η = 1, it behaves as a capacitor with *C* = *B*; when η = 0, it acts as a resistor with *R* = 1/*B*. Using Euler’s identity $j=e^{j\pi /2}=\cos\;\frac{\pi }{2}+j\sin\;\frac{\pi }{2}$ , the CPE impedance can be expressed as $Z_{\mathrm{CPE}}=\frac{1}{\omega^{\eta }B}\left[\cos\left(\eta \frac{\pi }{2}\right)-j\sin\left(\eta \frac{\pi }{2}\right)\right]$ with a phase angle is $\theta_{\mathrm{CPE}}=-\eta \frac{\pi }{2}$ . Nyquist and spectral plots illustrating the effect of η are provided in Supporting Information.

## 3 Materials and Methods

The experiment measures the electrical properties of water, isopropyl alcohol (isopropanol), and wet soil from 20 Hz to 1 GHz using an LCR meter and time domain reflectometry (TDR). Wet soil is unsaturated kaolinite with porosity ϕ of 0.27, gravimetric water content *w* = 0.35, degree of saturation *S*_*w*_ = 0.94 and volumetric water content θ_*w*_ = ϕ*S*_*w*_ = 0.26. Each sample type is measured for three times independently and each measurement collects three averaged LCR signals and 12 stacked TDR signals.

We use a two-electrode setup (impedance spectroscopy) instead of a four-electrode system (spectral induced polarization, SIP) because: (1) the TDR coaxial probe operates on a two-electrode principle, allowing a single probe for both TDR and LCR measurements, hereby, minimizing sample disturbance; and (2) the four-electrode method often introduces two artifacts: electrode polarization below 10 kHz^26,31,35^ and inductive electromagnetic coupling^35,46^ or parasitic capacitive coupling^32^ above 1 kHz.

### 3.1 Low-Frequency Measurements

We test water (to determine the geometric factor β), isopropanol, and wet kaolinite using two-electrode probes. The term impedance spectroscopy broadly refers to the measurement of a system’s impedance across a range of frequencies, and it can be applied using two-, three-, or four-electrode configurations. In the geoscience community, impedance spectroscopy is commonly associated with two-electrode setups. In contrast, the four-electrode configuration is linked to the spectral induced polarization (SIP) method. The use of three-electrode configurations is uncommon in geoscience but is standard practice in electrochemistry, catalysis, and battery research, where it is known as electrochemical impedance spectroscopy (EIS).^1^

Impedance spectroscopy is applied from 20 Hz to 2 MHz via an LCR meter (Keysight E4980A) at *V*_rms_ of 1 V. Probe materials (coaxial aluminum-graphene, stainless steel, or Ag/AgCl) are specified in figure captions. All measurements are performed at ambient pressure and room temperature. Each probe is calibrated (open/short) following established procedures.^11,41^ Figure 2 shows representative LCR data: impedance magnitude |Z*| and phase angle θ_*Z*_. Circuit modeling is used to extract intrinsic properties masked by artifacts such as electrode polarization. The fitting of measured impedance and circuit model is to achieve a minimal error based on L2-norm (least-square method).

**Figure 2:**
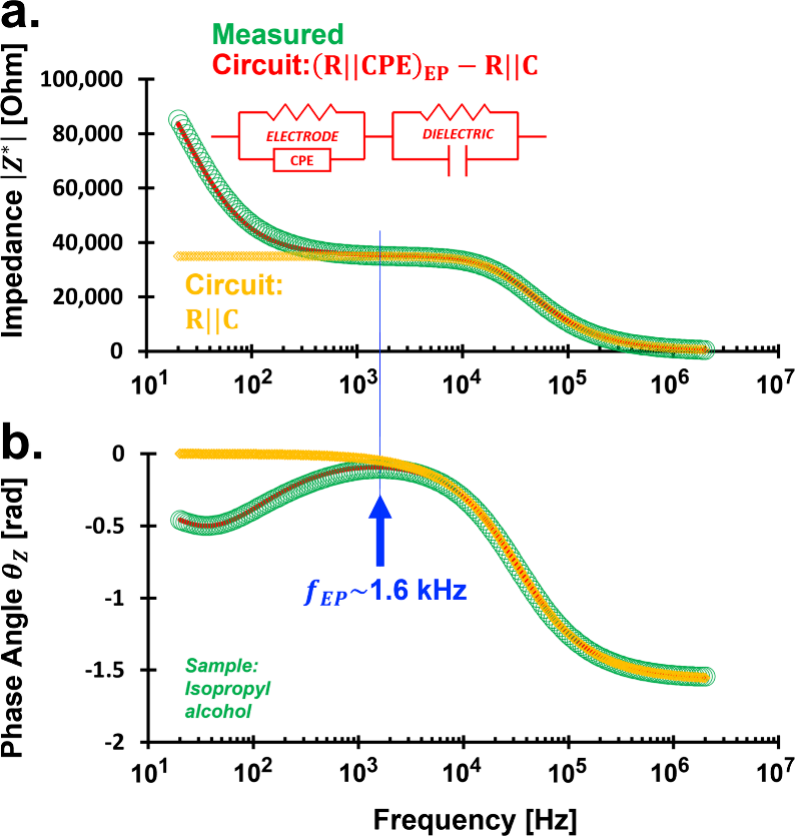
Bode plots and circuit modeling for isopropanol. Impedance magnitude |Z*| in Ohm and phase angle θ_*Z*_ in radians. Sample: isopropyl alcohol (isopropanol). Probe: coaxial aluminum-graphene, geometric factor β = 0.8 m. Configuration: Two-electrode. Blue arrow points out the electrode polarization (EP) limiting frequency *f*_EP_. Circuit model parameters: *R* = 35 kΩ, *C* = 137 pF, *R*_EP_ = 90 kΩ, *B*_EP_ = 180 nF Hz^1−η^, and η = 0.88.

### 3.2 High-Frequency Measurements

High-frequency spectra (1 MHz–1 GHz) are obtained using a time domain reflectometer (TDR; brand: Sympuls Aachen, series: TDR3000) and analyzed via the reflection decoupled ratio (RDR) method, detailed in recent studies.^11,41,47^ The estimation of permittivity is constrained by the least-square method (L2-norm) to obtain the least error between measured and predicted complex RDR function. High-frequency probe uses a BNC-to-UHF 50 Ω adapter (type: PL259-SO239) and the mismatch is a BNC-to-BNC 75 Ω adapter. This method estimates the complex relative permittivity κ*_eff_ from the frequency-domain signal ratio RDR = *R*_rem_/*R*_1_, derived from time-domain signals, *r*_rem_(*t*) and *r*_1_(*t*). Here, *r*_rem_(*t*) captures all remaining reflections from the sensing section, while *r*_1_(*t*) originates from the source and reflected by the mismatch section (different characteristic impedance between the leading cable and sensing section). Initial tests indicate that TDR-RDR performs well for liquids (1 MHz–1 GHz)^11^ and wet soils (1–100 MHz).

Using the simplified mixing model κ′ ≈ θ_*w*_κ_*w*_^′^, and assuming a pore fluid permittivity κ_*w*_^′^ of approximately 70–78 with a prepared volumetric water content θ_*w*_ of 0.26, the resulting saturated soil permittivity κ′ is estimated to be around 18–21. Isopropyl alcohol (κ_s_ ≈ 19) is therefore employed as a calibration reference due to the proximity of its value to wet soil. High-frequency spectra also aid to determine geometric factor β for low-frequency measurements, ensuring spectral continuity.^11,41^ We provide the RDR codes in the Supporting Information.

## 4 Results and Discussion

### 4.1 Proposed Circuit Models

#### 4.1.1 Inherent Material Polarizations

Materials exhibit both resistive and capacitive behaviors: they conduct charge (defining resistivity or conductivity) and store charge at pores, interfaces, phase boundaries, defects, or surfaces.^1^ This duality is commonly modeled as a parallel resistor–capacitor circuit (*R*∥*C*).^48,49^ To account for distributed resistances at charge storage sites, the capacitor can be replaced with a constant phase element (CPE), yielding the *R*∥CPE model. At low frequencies, increased electrode polarization thickens the electrical double layer *d*_EP_ at the electrode—material interfaces, leading to higher resistance *Z* ^′^, where *Z* ^′^ = *d*_EP_σ_eff,EP_^′^/*A*_probe_, assuming constant effective conductivity of polarized ions (σ_eff,EP_^′^) and probe surface area (*A*_probe_).

Table 1 summarizes the *Z*_Z_^*^ of various circuit models. The most general *R*∥CPE model can be derived as

$$\frac{1}{Z_{Z}^{*}}=\frac{1}{R}+\frac{1}{Z_{\mathrm{CPE}}}=\frac{1}{R}+(j\omega {)}^{\eta }B$$ 12

$$Z_{Z}^{*}=\frac{R}{1+(j\omega {)}^{\eta }RB}=\frac{R}{1+(j\omega \tau_{Z}{)}^{\eta }},\;\tau_{Z}=(RB{)}^{1/\eta }$$ 13

For η = 1, *Z*_Z_^*^ reduces to *Z*_∥_^*^ for the *R*∥*C* model with *B* = *C*:

$$Z_{∥}^{*}=\frac{R}{1+j\omega \tau_{∥}},\;\tau_{∥}=RC$$ 14

At low frequencies ω → 0, Z* → *R*; at high frequencies ω → ∞, Z* → 0. This results in a decreasing *Z*′ across a frequency sweep (Figure 3a).

**Figure 3:**
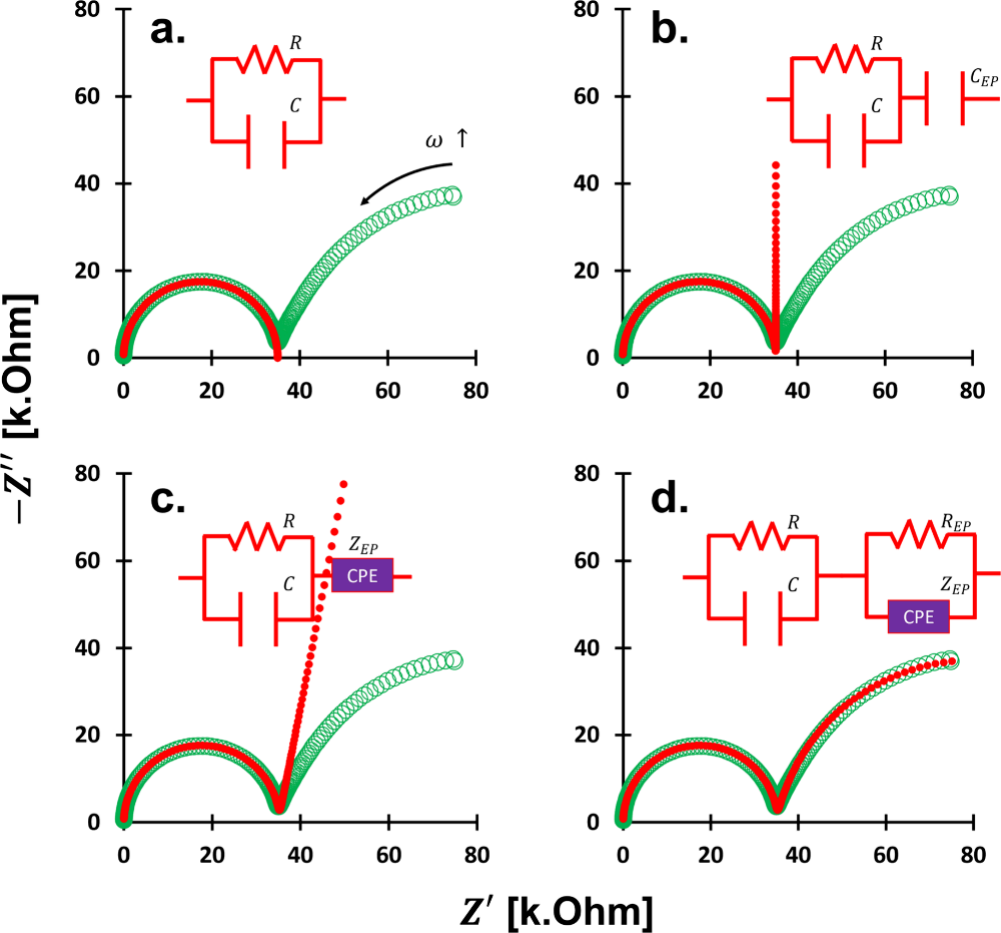
Nyquist plots and circuit modeling for isopropanol. Measured data in circle empty greens and model in red circle. Sample: isopropanol. Measured data same in Figure 2. Explored circuit models are on the electrode polarization: (a) No electrode polarization and only material polarization (*R* = 35 kΩ, *C* = 137 pF), (b) as a single capacitor (*C*_EP_ = 180 nF), (c) as a constant-phase-element CPE component (*B*_EP_ = 180 nF Hz^1−η^, η = 0.88), and (d) as a resistor and CPE in a parallel connection (*R*_EP_ = 90 kΩ, *B*_EP_ = 180 nF Hz^1−η^, η = 0.88).

The capacitance is geometrically defined as *C* = ε_s_(*A*/*L*) = ε_s_β, and becomes frequency-dependent as *C* = ε*β at varying frequencies. Given a DC conductivity, σ_DC_ = (*R*β)^−1^, the complex admittance *Y*_∥_^*^ = 1/*Z*_∥_^*^ for the *R*∥*C* circuit model is

$$Y_{∥}^{*}=\frac{1}{R}+j\omega C=\beta \sigma_{\mathrm{DC}}+j\omega \beta \varepsilon^{*}=\omega \beta \frac{\sigma_{\mathrm{DC}}}{\omega }+j\omega \beta (\varepsilon^{\prime }-j\varepsilon^{\prime \prime })=\omega \beta \varepsilon_{\mathrm{eff}}^{\prime \prime }+j\omega \beta \varepsilon^{\prime }$$ 15

Using Y* = *Y*′ + *jY*″ and $\varepsilon_{\mathrm{eff}}^{\prime \prime }=\frac{\sigma_{\mathrm{DC}}}{\omega }+\varepsilon^{\prime \prime }$ , eq 15 yields^26,50^

$$\{\begin{array}{c} \varepsilon^{\prime }=\frac{Y^{\prime \prime }}{\beta \omega }=\frac{1}{\beta \omega }|Y^{*}|\sin\;\theta_{Y}=\frac{-1}{\beta \omega }\frac{\sin\;\theta_{Z}}{\left|Z^{*}\right|}\\ \varepsilon_{\mathrm{eff}}^{\prime \prime }=\frac{Y^{\prime }}{\beta \omega }=\frac{1}{\beta \omega }|Y^{*}|\cos\;\theta_{Y}=\frac{1}{\beta \omega }\frac{\cos\;\theta_{Z}}{\left|Z^{*}\right|} \end{array}$$ 16

where admittance and impedance phase angles are related by θ_*Y*_ = −θ_*Z*_. Notably, the real component: $\varepsilon^{\prime }=\frac{Y^{\prime \prime }}{\beta \omega }=\frac{C}{\beta }$ or $\kappa^{\prime }=\frac{C}{\beta \varepsilon_{0}}=\frac{C}{C_{0}}$ ; while the effective imaginary component: $\varepsilon_{\mathrm{eff}}^{\prime \prime }=\frac{Y^{\prime }}{\beta \omega }$ or $\kappa_{\mathrm{eff}}^{\prime \prime }=\frac{1}{\beta R\omega \varepsilon_{0}}=\frac{\sigma_{\mathrm{DC}}}{\omega \varepsilon_{0}}$ and eq 16 also yields *ε*_eff_^*^ without assuming a specific circuit model (cf. eq 6).

The *R*∥*C* circuit provides the simplest framework to recover ε_eff_^*^ and represent polarization effects. However, ε_eff_^*^ can also be derived directly from measurements, potentially capturing multiple polarization mechanisms and artifacts (e.g., electrode polarization, inductive coupling), as the number of contributing processes is unknown.

For the *R*∥CPE model, the complex admittance is

$$Y_{Z}^{*}=\frac{1+(j\omega \tau_{Z}{)}^{\eta }}{R}=\frac{1+\omega^{\eta }\tau_{z}^{\eta }\cos\left(\eta \frac{\pi }{2}\right)}{R}+j\frac{\omega^{\eta }\tau_{z}^{\eta }\sin\left(\eta \frac{\pi }{2}\right)}{R}$$ 17

and the subsequent complex permittivity ε_eff_^*^ is

$$\{\begin{array}{l} \varepsilon^{\prime }=\frac{Y^{\prime \prime }}{\beta \omega }=\frac{B\sin\left(\eta \frac{\pi }{2}\right)}{\beta \omega^{1-\eta }}\\ \varepsilon_{\mathrm{eff}}^{\prime \prime }=\frac{Y^{\prime }}{\beta \omega }=\frac{1}{\beta R\omega }+\frac{B\cos\left(\eta \frac{\pi }{2}\right)}{\beta \omega^{1-\eta }} \end{array}$$ 18

According to eq 18, the real permittivity ε′ decreases with frequency following the negative slope ( $1-\tilde{\eta }$ ) in a log–log plot:

$$-\frac{\partial \log\;\kappa^{\prime }}{\partial \log\;f}=1-\tilde{\eta }$$ 19

Here, $\tilde{\eta }$ is an effective CPE exponent. For a single *R*∥CPE model, $\tilde{\eta }=\eta$ . In circuits comprising multiple *R*∥CPE elements, the slope becomes ( $1-\tilde{\eta }$ ) or ( $1+\tilde{\eta }$ ) (see Supporting Information). The value of $\tilde{\eta }$ is frequency-dependent and influenced by the dominant circuit components operating within a specific frequency regime.

For ideal dielectrics (no dispersion), a flat permittivity spectrum yields in $\frac{\partial \log\;\kappa^{\prime }}{\partial \log\;f}=0$ and $\tilde{\eta }=1$ , meaning an ideal capacitor. In contrast, highly conductive materials often exhibit dominant electrode polarization, characterized by a slope of −1 and $\tilde{\eta }=0$ , indicating resistive interfacial behavior. At low frequencies, where conduction prevails, ε_eff_^″^ exhibits a slope of −1 primarily governed by the $\frac{1}{\beta R\omega }$ term, which outweighs the contribution from the dispersive component $\frac{B\cos\left(\eta \frac{\pi }{2}\right)}{\beta \omega^{1-\eta }}$ .

#### 4.1.2 Electrode Polarizations

Electrode polarization arises from charge accumulation at the material–electrode interfaces, where no charge transfer occurs.^1^ This leads to a voltage drop due to static or diffusing ions within the electrical double layer, typically a few nanometers thick *d*_EP_.^6,51^ This interface can be modeled as *C*, CPE, *R*∥*C*, or *R*∥CPE. The total measured impedance *Z*_t_^*^ is the sum of inherent material polarization *Z*_m_^*^ and electrode polarization artifacts *Z*_EP_^*^:^6,52^

$$Z_{t}^{*}=Z_{m}^{*}+Z_{\mathrm{EP}}^{*}$$ 20

Sensitivity analyses in Supporting Information show that electrode polarization consistently manifests at low frequencies.

### 4.2 Cole–Cole vs Circuit Model: Isopropyl Alcohol

The measurement system is reliable above 1.6 kHz, defined as the electrode polarization limiting frequency *f*_EP_, below which prevailing electrode polarization (EP) effects distort the material response (Figure 2). In this regime (*f* < *f*_EP_), the reduced ionic mobilities at material–electrode interfaces hinder conductivity (Figure 2a).

We apply an *R*∥*C* model for the material polarization impedance *Z*_m_^*^ and an *R*∥CPE model for the electrode polarization *Z*_EP_^*^, with a reasonable fit, confirmed by a Nyquist plot (Figure 3a). From this, the true complex permittivity ε_eff_^*^ is derived. The corrected phase angle θ_*Z*_ approaches zero (Figure 2b), resulting in a nearly constant permittivity κ′ ≈ κ_s_.

The dielectric constant is calculated as κ_s_ = *C*/*C*_0_ = *C*/(ε_0_β), yielding 19.35, which aligns with the literature value used in the Cole–Cole model (κ_s_ = 19.34,^11^ *f* < 50 MHz; Figure 4). Within this range, the material behaves as a dielectric or insulator. Meanwhile, the DC conductivity is estimated from resistance: σ_DC_ = (β*R*)^−1^ = (0.8 m × 35 kΩ)^−1^ ≈ 3.57 × 10^–5^ S/m, consistent with in-phase conductivity σ_eff_^′^ = ωε_eff_^″^ ≈ 3.50 × 10^–5^ S/m, obtained from Cole–Cole fitting over 300 Hz–1 MHz (Figure 4). As σ_eff_^′^ = 2π*f*ε_0_κ_eff_^″^, the slope of $\left(\log\;\kappa_{\mathrm{eff}}^{\prime \prime }=\log\;\frac{\sigma_{\mathrm{DC}}}{2\pi \varepsilon_{0}}-\log\;f\right)$ is −1 in the conduction regime (σ_eff_^′^ ≈ σ_DC_).

**Figure 4:**
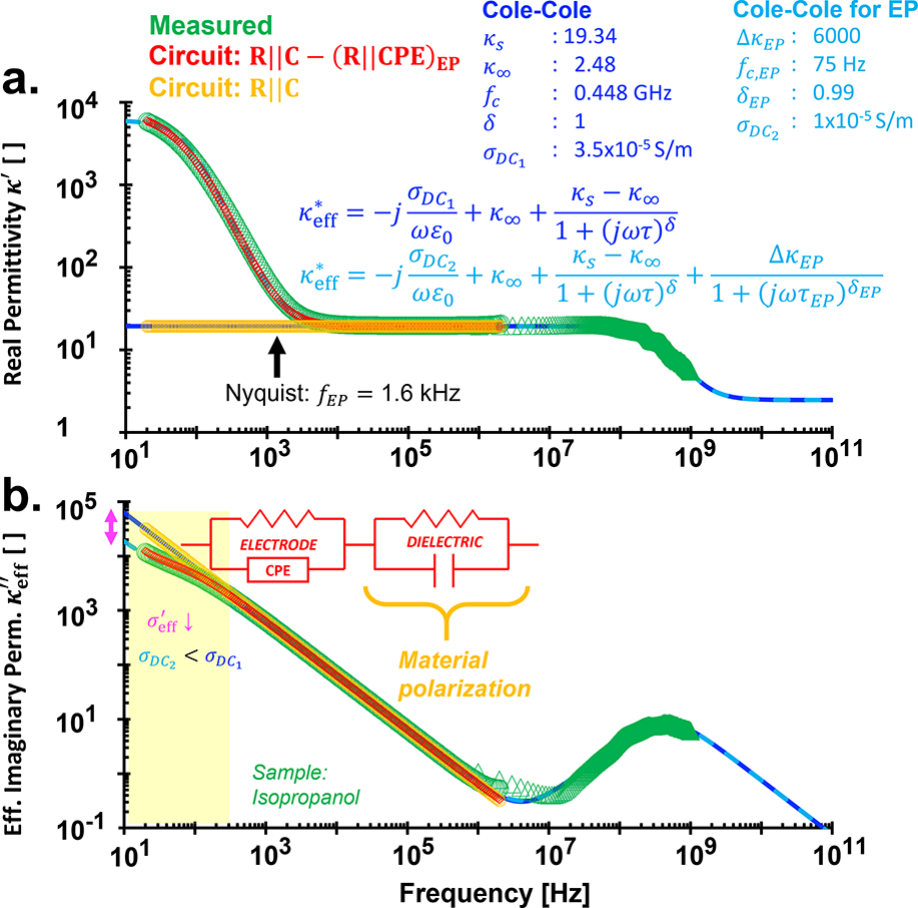
Complex relative permittivity spectra κ_eff_^*^ of isopropanol (isopropyl alcohol): (a) Real relative permittivity κ′ and (b) effective imaginary relative permittivity κ_eff_^″^. The electrode polarization limiting frequency *f*_EP_ is at 1.6 kHz. See also Figure S6 in the Supporting Information.

We show that both Cole–Cole and circuit model (*R*∥*C* – CPE) yield nearly identical numerical fits across the entire measured permittivity spectrum of isopropanol, capturing both electrode and inherent material polarizations (Figure 4; see also Figure S6 in Supporting Information). The Cole–Cole model used here is the extended version with multiple relaxation terms (eq 11). Notable, incorporating electrode polarization reduces the estimated DC conductivity from 3.5 to 1 × 10^–5^ S/m (σ_DC_1__ > σ_DC_2__ in Figure 4).

In the Cole–Cole model, the upper bound of relative permittivity due to electrode polarization (Δκ_EP_) is initially estimated visually from the spectrum and subsequently optimized to minimize the permittivity fitting error. In contrast, the circuit model fits the complex impedance Z* in the Nyquist domain, from which the corresponding permittivity spectra are derived. The circuit model generates permittivity spectra with and without accounting for electrode polarization. The validity of the permittivity response without electrode polarization is evident, as isopropanol, a low-viscosity liquid, predominantly exhibits orientational polarization.

Increasing the thickness of the material under test can, in principle, increase the electrode polarization relaxation time τ_EP_ in the Cole–Cole model^53^ (i.e., shifting the *f*_c,EP_ and its associated peak to lower frequencies). However, electrode polarization is inevitable, and increasing sample thickness is not always a practical solution. It is important to emphasize that the electrode polarization limiting frequency (*f*_EP_) is not equivalent to the critical frequency of electrode polarization (*f*_c,EP_ = 1/2πτ_EP_); they represent distinct physical properties (see Figure 4). Nevertheless, materials with lower *f*_c,EP_ generally exhibits correspondingly lower *f*_EP_.^11^

The Cole–Cole model enables the estimation of static permittivity κ_s_ below *f*_EP_, thereby validating the permittivity values derived from the circuit model. In the frequency range of 2 MHz–1 GHz, permittivity obtained using the TDR-RDR method is directly derived from the wave theory, eliminating the need for impedance conversion. However, if the complex impedance Z* is needed, it can be computed from the effective permittivity ε_eff_^*^ using eq 5.

Slope analysis in Figure 5a supports model selection: above 10 kHz, κ′ is constant (−*∂*log κ′/*∂*log *f* = 0), indicating $\tilde{\eta }=1$ (ideal capacitor in *R*∥*C*). Below 300 Hz, strong electrode polarization reduces σ_eff_^′^, causing κ_eff_^″^ deviations from both Cole–Cole and circuit models (Figure 4). The inflection at 300 Hz corresponds to the peak slope in –*∂*log κ′/*∂*log *f*, marking the electrode polarization-dominated regime (Figure 5a).

**Figure 5:**
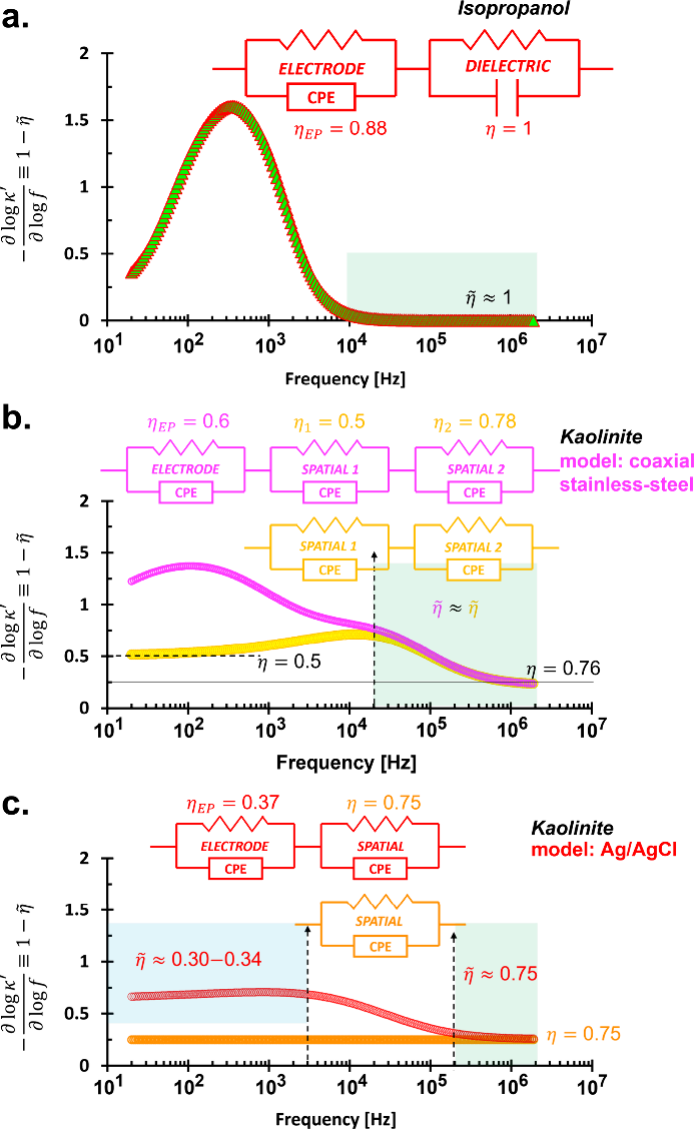
Permittivity slope spectra. It assists to analyze the effective CPE exponent $\tilde{\eta }$ . (a) Isopropyl alcohol, (b) wet kaolinite measured by stainless steel, and (c) wet kaolinite measured by Ag/AgCl. All data presented are from the circuit model. Insets are detailed circuit models.

We recognized that, in addition to the Debye and Cole–Cole models, several other models are widely used in the dielectric spectroscopy community. These include the Cole–Davidson model:^54^

$$\varepsilon^{*}=\varepsilon_{\infty }+\frac{\varepsilon_{s}-\varepsilon_{\infty }}{(1+j\omega \tau {)}^{\delta_{\mathrm{CD}}}}$$ 21

and the Havriliak–Negami model:^55^

$$\varepsilon^{*}=\varepsilon_{\infty }+\frac{\varepsilon_{s}-\varepsilon_{\infty }}{[1+(j\omega \tau {)}^{\alpha_{\mathrm{HN}}}{]}^{\beta_{\mathrm{HN}}}}$$ 22

The Havriliak–Negami model is a generalized formulation that incorporates features of both the Cole–Cole and Cole-Davidson models. It reduces to the Cole–Cole model when the outer power factor β_HN_ = 1 (in this case α_HN_ = δ), and to the Cole-Davidson model when the inner power factor α_HN_ = 1 (in which case β_HN_ = δ_CD_). The parameter α_HN_, also known as the spreading or broadening factor, originates from the Cole–Cole model and introduces symmetric broadening around the Debye peak, which is centered at the characteristic polarization frequency *f*_c_.

In practice, many alcohol-based liquids exhibit asymmetric dielectric dispersion, often characterized by a relaxation tail extending toward frequencies higher than *f*_c_.^56–58^ This behavior is typically attributed to relaxation processes associated with intermolecular networks formed by aligned polar molecules, often involving hydrogen bonding. These networks can give rise to a secondary relaxation peak to the primary Debye peak. Due to their proximity, the two processes may overlap, resulting in a broadened or skewed single peak. This apparent peak can be effectively modeled using the Cole-Davidson or Havriliak–Negami formulations.^58^

Unfortunately, it is not feasible to construct a lumped-element circuit model that replicates the form of the Cole–Davidson and Havriliak–Negami models, due to their inherently frequency-dependent characteristics. The experimental data obtained from the TDR-RDR method are limited to a frequency range up to 1 GHz, while the characteristic Debye relaxation frequency *f*_c_ is approximately 0.5 GHz. Thus, only a limited portion of the 0.5–1 GHz range is available for analysis (Figure 4). Moreover, the nonsymmetrical ε″-peak, caused by the high-frequency tail effect, is commonly observed around 6.8 GHz. This arises from a secondary relaxation process with a characteristic time of 23.4 ps at 25 °C.^56^ Given these constraints, the Cole–Cole model is considered practically sufficient for the scope of this study.

### 4.3 Cole–Cole vs Circuit Model: Wet Soil

Impedance Z*, an extrinsic (size-dependent) property, is typically normalized to intrinsic quantities such as complex conductivity σ_eff_^*^ or resistivity ρ_eff_^*^ using the geometric factor β. Figure 6 reveals that electrode polarizations influence the measured resistivity in wet kaolinite. Ideally, both coaxial stainless-steel and Ag/AgCl probes should yield consistent conductivity curves. Ag/AgCl is preferred due to minimal polarization.^32^ Spatial polarization may also distort Nyquist plots, complicating interpretation.

**Figure 6:**
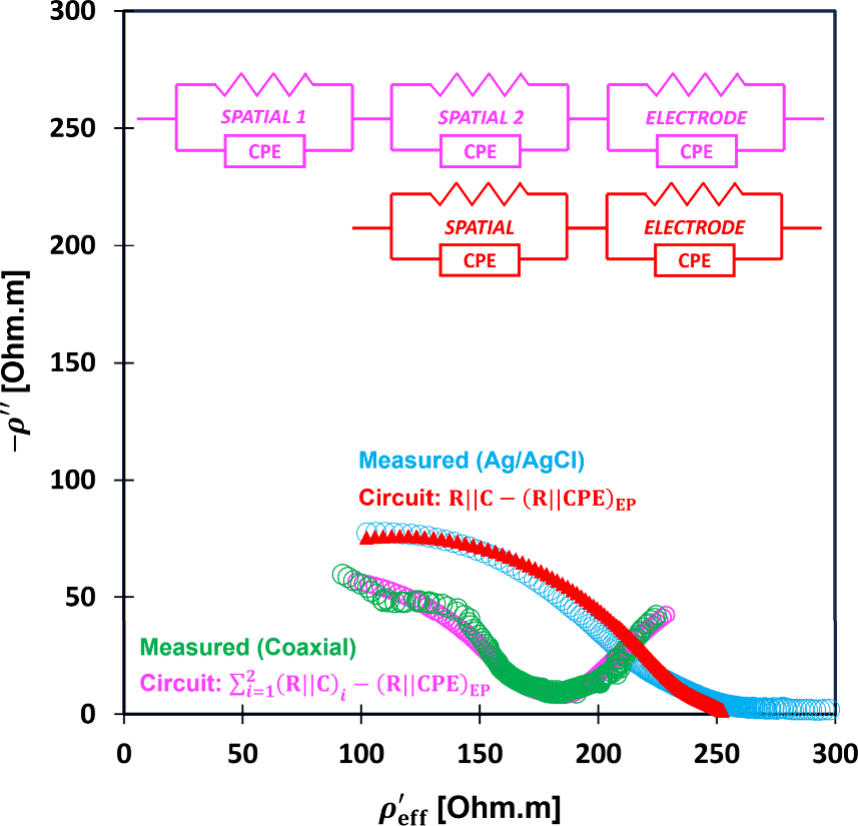
Circuit modeling for wet soil (kaolinite). Nyquist plot is also representable with – ρ″ vs ρ_eff_^′^ instead of the conventional – *Z*″ vs *Z*′ because ρ_eff_^*^ = βZ* and the geometric factor β is a constant real number: β_coaxial_ = 0.08 m and β_Ag/AgCl_ = 0.03 m. Detailed circuit parameters for the coaxial stainless-steel probe: *R*_1_ = 375 Ω, *B*_1_ = 3 × 10^–3^ F Hz^1−η^, η_1_ = 0.5, *R*_2_ = 2 kΩ, *B*_2_ = 2.1 × 10^–6^ F Hz^1−η^, η_2_ = 0.78, *R*_EP_ = 4.5 kΩ, *B*_EP_ = 3.1 × 10^–1^ F Hz^1−η^, η_EP_ = 0.6; Ag/AgCl probe: *R* = 7333 Ω, *B* = 5.6 × 10^–6^ F Hz^1−η^, η = 0.75, *R*_EP_ = 1167 Ω, *B*_EP_ = 1.5 × 10^–2^ F Hz^1−η^, η_EP_ = 0.37.

In wet soils, the Cole–Cole model may not capture real permittivity κ′ accurately below the electrode polarization limiting frequency *f*_EP_ as there is no benchmark value to constrain how high the spatial polarization effects could be (unknown Δκ_spatial_=Δε_spatial_/ε_0_). Thus, we assume the model remains constant below 100 kHz (Figure 7a-i,b-i). While spatial effects may exist from 20 Hz to 100 kHz but they cannot be isolated from electrode polarization. Meanwhile, circuit models that exclude electrode polarization effects *Z*_EP_^*^ are utilized to transform the true material’s complex impedance *Z*_m_^*^ and retrieve the intrinsic permittivity spectra.

**Figure 7:**
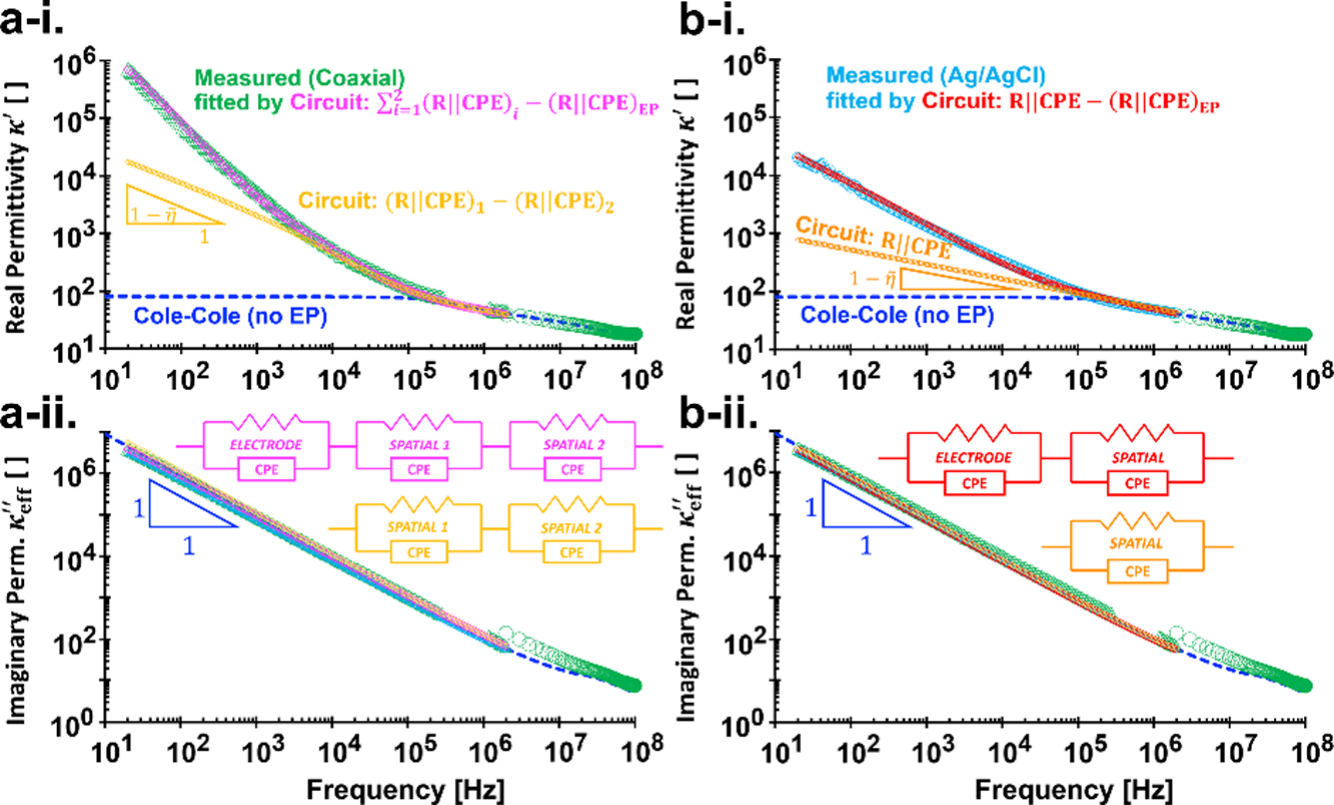
Complex relative permittivity spectra κ_eff_^*^ of wet soil (kaolinite). (a) (left column) Circuit model is to fit the data measured with coaxial TDR probe. (b) (right column) Circuit model is to fit the data measured with Ag/AgCl probe. Detailed legends are inside the real permittivity plots (i) and the circuit elements appear as insets inside the effective imaginary permittivity plots (ii). Circuit model parameters are in the caption of Figure 6. Cole–Cole parameters are specified in the caption of Figure 8. Slope signs for the real permittivity is ( $1-\tilde{\eta }$ ), where $\tilde{\eta }$ is a frequency-dependent effective CPE exponent.

The electrode polarization circuit elements *R*_EP_ and CPE_EP_ vary between probes due to differences in ion affinity at the electrode-material interface. Stainless-steel shows higher resistance than Ag/AgCl (*R*_EP_SS__ > *R*_EP_Ag/AgCl__). This suggests a thicker polarization layer *d*_EP_ which impedes ionic mobility and increases voltage drop. However, *d*_EP_ cannot be accurately estimated due to unknown electrode spacing in irregular geometries. It is defined as *d*_EP_ = κ′ε_0_ *A*_probe_/*C*_EP_.^6,51^

The two probes produce different polarization resistivities ρ_eff_^*^ due to less cable resistances on the stainless-steel system compared to that of Ag/AgCl (Figure 6). We do not eliminate the ρ_eff_^′^ from cable resistances to avoid overlapping plots (all Nyquist plots of resistivity are supposed to emerge in the same curve). However, their real permittivity κ^′^ converges above respective *f*_EP_ (10^4^–10^5^ Hz in Figure 7a-i,b-i). Both probes also exhibit similar κ_eff_^″^ over the full frequency range (Figure 7a-ii,b-ii).

Derived resistivities are 190 Ω m (=β*R*_1_ + β*R*_2_) for stainless-steel and 220 Ω m for Ag/AgCl, corresponding to DC conductivities of 5.3 and 4.6 mS/m, respectively. Both values are consistent with the Cole–Cole model estimate of 5.0 mS/m, supporting the observed unified κ_eff_^″^ behavior.

Both Cole–Cole and circuit models also converge for κ_eff_^″^. As shown in eq 18, the term $\frac{(\beta R{)}^{-1}}{\omega }$ dominates over ω^η–1^, since it has a steeper slope, i.e., −1 in log–log plots. Thus, $\kappa_{\mathrm{eff}}^{\prime \prime }\approx \frac{\sigma_{\mathrm{DC}}}{\varepsilon_{0}\omega }$ across the LCR frequency range.

For stainless-steel, κ′ converges above 2 × 10^4^ Hz (Figure 7a-i), with spatial polarization indicated by a minor peak near 2 × 10^4^ Hz (η_peak_ ≈ 0.3) and electrode polarization by a stronger peak at 100 Hz (η_EP_ = 0.6). Effective CPE exponents vary between $\tilde{\eta }$ ≈ 0.5–0.76 (Figure 5b), resulting from two (*R*∥CPE) models with η_1_ = 0.78 and η_2_ = 0.5.

Similar trends are observed for Ag/AgCl (Figure 7b-i), with κ′ convergence above 2 × 10^5^ Hz, influenced by spatial polarization. The slope plot (Figure 5c) indicates η = 0.75 postcorrection, with stable precorrection slopes $\tilde{\eta }$ ≈ 0.30–0.34 (below 3 kHz) and $\tilde{\eta }$ ≈ 0.75 (above 200 kHz), corresponding to η_EP_ = 0.37 and spatial η = 0.75. Between 3 and 200 kHz, the slope exhibits dispersive behavior due to overlapping CPE effects.

Finally, the choice of plotting domain, whether complex conductivity σ_eff_^*^, resistivity ρ_eff_^*^, or relative permittivity κ_eff_^*^, influences numerical sensitivity and interpretive clarity. Electrode polarization, in particular, can obscure accurate estimation of intrinsic, probe-independent DC conductivity.

### 4.4 Electric vs Hydraulic Properties

TDR-RDR measurements at 100 MHz yield measured permittivity of κ′ = 18.2 for the wet kaolinite sample and κ_w_ = 50 for the supernatant water (much lower than that of fresh water permittivity ∼80 due to dissolved salts). Assuming a mineral permittivity κ_m_ is 7, a degree of saturation *S*_w_ = 0.94–1 (the actual saturation from density and water content measurements is 0.94), known air permittivity κ_a_ = 1, We estimate the porosity ϕ of soil using a permittivity mixing model:^1^ κ′ = ϕ*S*_w_κ_w_ + (1 – ϕ)­κ_m_ + ϕ­(1 – *S*_w_)­κ_a_. Based on this model, the predicted porosity ϕ_pred_ ranges from 0.26 to 0.28, depending on the assumed saturation. This prediction aligns well with the actual measured porosity of 0.27. The range of predicted values reflects the uncertainty in saturation.

We then examine the hydraulic properties from the DC conductivity of saturated soil (σ_soil_) and supernatant water (σ_w_). Assuming the wet soil is fully saturated (*S*_w_ = 1), a simplified conductivity mixing model (Archie’s equation in ref 13) predicts porosity as ϕ_pred_ = σ_soil_/σ_w_ = (5.0 mS/m)/(53 mS/m) = 0.1. This estimation is significantly under prediction of the actual porosity of 0.27. When incorporating a cementation factor *m*_c_ which typically ranges from 1.55 to 2.11 for clayey soils and rocks,^13,59^ the predicted porosity becomes ϕ_pred_ = (σ_soil_/σ_w_)^1/*m*_c_^, yielding values in the range of 0.23–0.30. These predictions better align with the actual porosity, highlighting the importance of considering a cementation factor *m*_c_ in conductivity-based porosity estimations for clayey particulate and porous media.

### 4.5 Spatial Polarizations in kHz–sub-GHz

The preceding section on wet soil examined the use of the Cole–Cole and equivalent circuit models to infer permittivity values across the full frequency range of 20 Hz–0.1 GHz. As previously noted, there is no definitive benchmark for identifying true spatial polarization effects, as these often coexist with electrode polarization in low-frequency measurements.^1^ In this section, we aim to isolate potential spatial polarization effects within the frequency range of 10^4^–10^8^ Hz (Figure 8a,b), where such artifacts are significantly reduced at frequencies exceeding the electrode polarization limiting frequency (*f* ≥ *f*_EP_).

**Figure 8:**
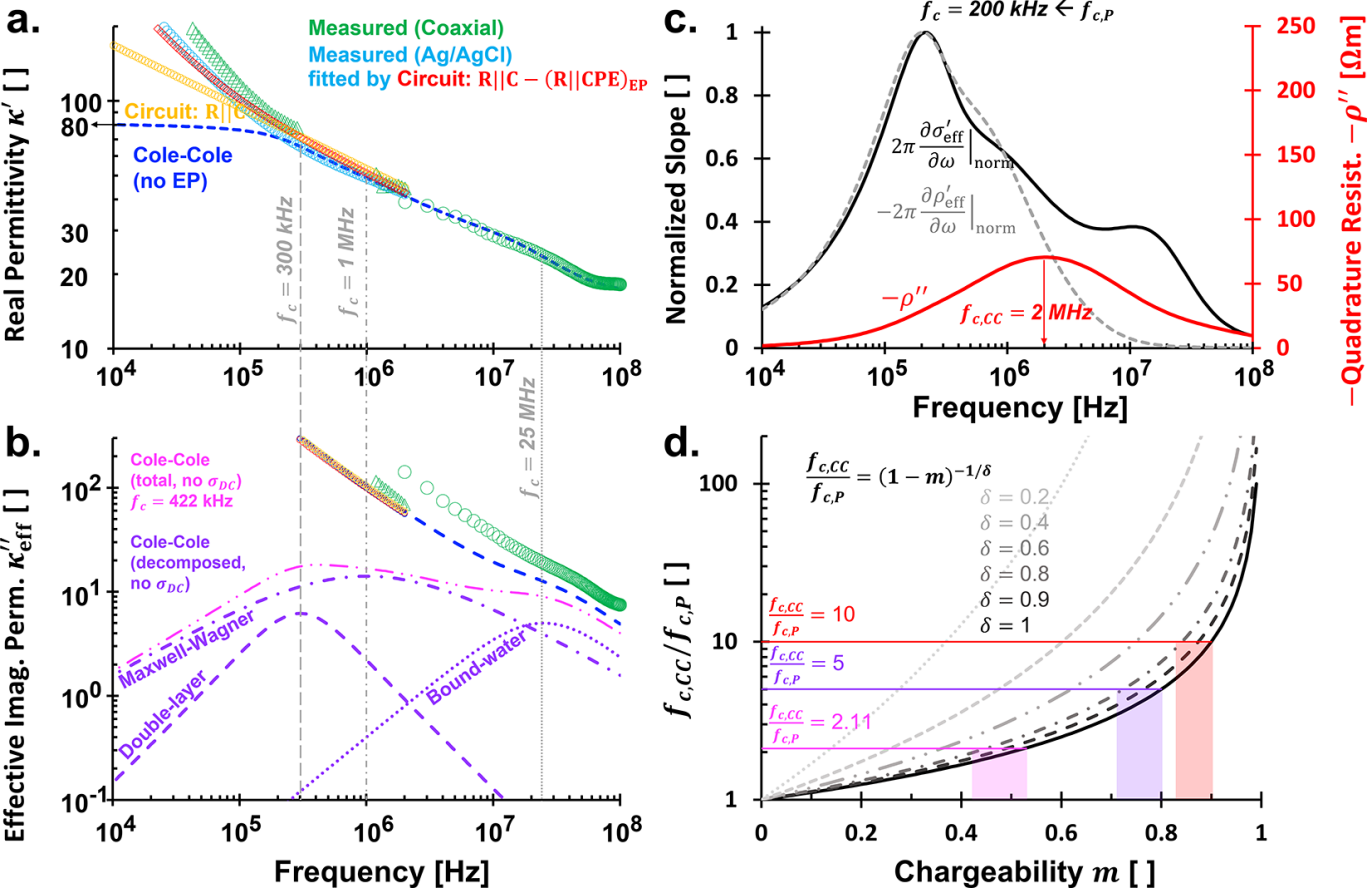
Cole–Cole model and chargeability analyses on wet soil (kaolinite). Real and imaginary relative permittivity (a,b). Frame (a) is a zoomed version of Figure 7b-i. Detailed Cole–Cole parameters for conduction: σ_DC_ = 5 × 10^–3^ S/m; orientational polarization: κ_∞_ = 2.2, κ_s_ = 15, *f*_c_ = 18 GHz, δ = 1; spatial polarizations in bigger scales: κ_∞_ = 0, κ_s_ = 9, *f*_c_ = 300 kHz, δ = 1.2; spatial polarizations in smaller scales: κ_∞_ = 0, κ_s_ = 46, *f*_c_ = 1 MHz, δ = 0.7; and spatial polarizations due to bound-water: κ_∞_ = 1, κ_s_ = 11, *f*_c_ = 25 MHz, δ = 1. (c) Normalized slope of effective real conductivity, resistivity, and the actual values of imaginary resistivity. Normalization refers to division by its maximum value. (d) Effects of chargeability *m* and Cole–Cole exponent δ onto critical frequency ratio between Cole–Cole and Pelton’s models. Highlighted ratios: *f*_c,CC_/*f*_c,P_ = 2 MHz/200 kHz = 10 (red), 1 MHz/200 kHz = 5 (purple), and 422 kHz/200 kHz = 2.11 (pink).

Spatial polarization spectroscopy reveals three critical frequencies, approximately 300 kHz, 1 MHz, and 25 MHz, which may correspond to double-layer, Maxwell–Wagner, and bound-water polarization mechanisms, respectively. Double-layer polarization involves the rearrangement of counterions within the electrical double layer surrounding clay particles.^1^ In contrast, Maxwell–Wagner polarization arises from charge accumulation at interfaces between materials with differing electrical properties, such as structural boundaries or trapped fluid phases.^1^ While both Maxwell–Wagner and double-layer polarizations contribute to interfacial polarization and may overlap or swap position in frequency depending on the system, they originate from distinct mechanisms: double-layer polarization arises from electrochemical phenomena at charged interfaces, whereas Maxwell–Wagner polarization results from permittivity and conductivity contrasts between bulk phases.^1^

As polarization scale gets larger, relaxation time τ lengthens, and the critical frequency *f*_c_ decreases. These phenomena are governed by ion diffusion within space, wherein the characteristic length *L*_c_ and dielectric relaxation times τ are related to the ionic diffusion coefficient *D* as follows:^1,29^

$$D=\frac{L_{c}^{2}}{\tau }$$ 23

Several studies have investigated the most suitable geometric parameter representing the characteristic length *L*_c_ in porous and particulate media, considering either pore^16,17,60–62^ or grain size.^63–65^ Additionally, some studies propose a pore-size dependent diffusion coefficient *D* = *D*(*r*), which extends eq 23 to predict pore size.^17,61,62^

### 4.6 Chargeability and Relaxation Time

Material scientists and chemists often express intrinsic electric properties using the effective complex permittivity ε_eff_^*^ or electrical moduli *M*_eff_^*^ = 1/ε_eff_^*^, whereas geophysicists typically employ equivalent representations such as complex conductivity σ_eff_^*^ or resistivity ρ_eff_^*^. A detailed derivation of the interrelationships among permittivity, conductivity, resistivity spectral functions, and their respective relaxation times is provided in Supporting Information.

We can express a conductivity model in an analogous form to the Cole–Cole permittivity model by replacing ε_∞_ with σ_∞_ and ε_s_ with σ_0_. However, these replacements are not dimensional conversions; specifically, σ_∞_ ≠ ωε_∞_ and σ_0_ ≠ ωε_s_. Importantly, σ_0_ represents the DC conductivity, σ_DC_ = ωε_eff,ω≈0_^″^; whereas ε_s_ corresponds to the real part of permittivity and not to *ε*_eff,ω≈0_^″^. These parameters (ε_s_, ε_∞_, σ_0_, σ_∞_) are each distinctly observable in the real-part spectrum of permittivity ε′ and effective conductivity σ_eff_^′^.

Consequently, the Cole–Cole model can be reformulated into a complex conductivity form,^39,40^ denoted σ_CC_^*^, which is analogous to eq 8 and can also incorporate chargeability *m*, as derived in Supporting Information:

$$\sigma_{\mathrm{CC}}^{*}=\sigma_{\infty }+\frac{\sigma_{0}-\sigma_{\infty }}{1+(j\omega \tau_{\mathrm{CC}}{)}^{\delta }}=\sigma_{0}\left[1+\frac{m}{1-m}\left[1-\frac{1}{1+(j\omega \tau {)}^{\delta }}\right]\right]$$ 24

Here, chargeability is fundamentally defined as $m=\frac{\sigma_{\infty }-\sigma_{0}}{\sigma_{\infty }}=\frac{\rho_{0}-\rho_{\infty }}{\rho_{0}}$ ^66^ (Figure 9a). Note: Cole-Cole relaxation times for conductivity τ_σ,CC_ and permittivity τ_ε,CC_ are not the same, τ_σ,CC_ ≠ τ_ε,CC_. They are only in analogous forms. For the simplicity, we write τ_σ,CC_ as τ_CC_ in eq 24, and τ_ε,CC_ as τ in eq 8. The writing of τ in permittivity models (Debye, Cole-Cole, Cole-Davidson, and Havriliak−Negami) is to emphasize the same Debye peak observed at *f_c_* = 1/2πτ.

**Figure 9:**
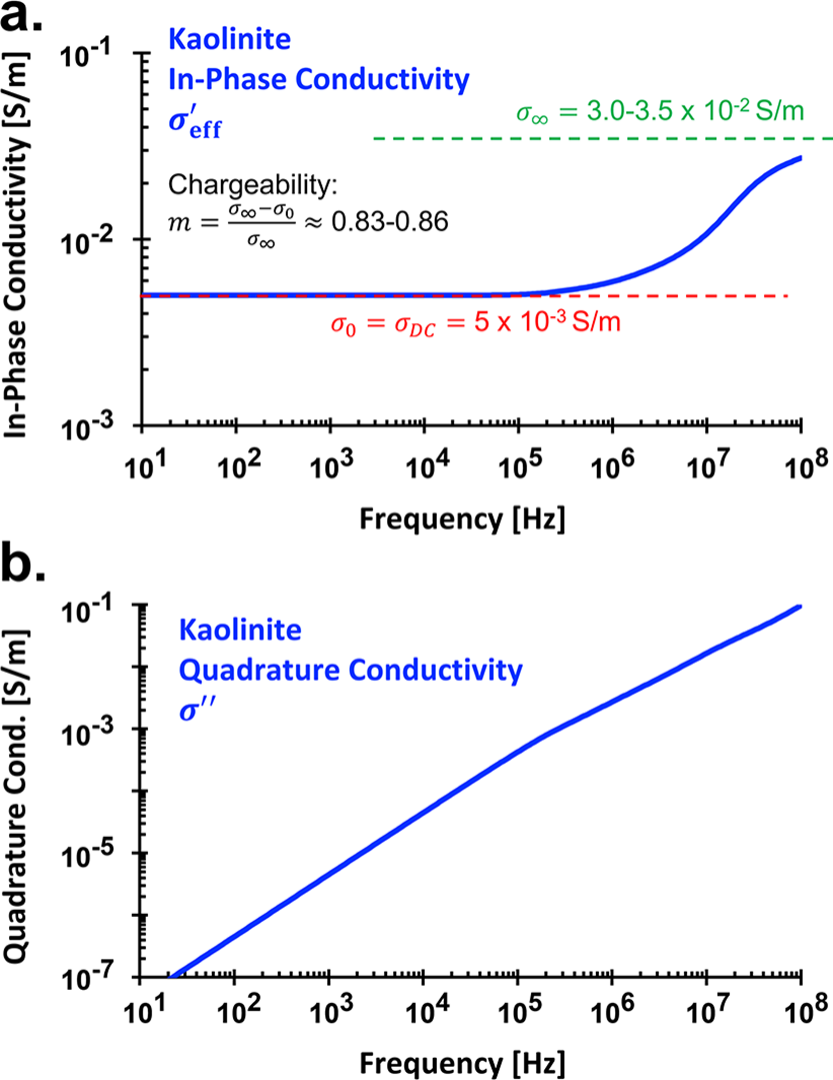
Complex conductivity spectra of wet kaolinite: (a) effective in-phase conductivity σ_eff_^′^ and (b) quadrature conductivity σ″. Full expression: σ_eff_^*^ = σ_eff_^′^ + jσ″.

The original Cole–Cole-type model introduced by Pelton, expressed in resistivity form ρ_P_^*^ and its conductivity equivalent (σ_P_^*^ = 1/ρ_P_^*^) are given by^39,40,46^

$$\rho_{P}^{*}=\rho_{\infty }+\frac{\rho_{0}-\rho_{\infty }}{1+(j\omega \tau_{P}{)}^{\xi }}\to \sigma_{P}^{*}=\sigma_{0}\left[1+\frac{m}{1-m}\times \left(1-\frac{1}{1+(j\omega \tau_{P}{)}^{\xi }(1-m)}\right)\right]$$ 25

The complex resistivity ρ_P_^*^ can be derived from the circuit model, corrected for electrode polarization using eq 13, such that ρ_P_^*^ = β*Z*_m_^*^. The parameters used in the impedance model *Z*_m_^*^ are identical to those in ρ_P_^*^.

From eq 13, the characteristic relaxation time of the impedance model is given by τ_*Z*_ = (*RB*)^1/η^, where η corresponds to ξ in eq 25, implying τ_*Z*_ = τ_P_. In our study, τ_*Z*_ is computed as 1.42 × 10^–2^ s and is associated with a spatial polarization (measured with Ag/AgCl electrodes). This corresponds to Pelton’s critical frequency, calculated as *f*_c,P_ = 1/(2πτ_*Z*_)= 11.23 Hz. This frequency lies below the limiting frequency for electrode polarization (*f*_EP_ ≈ 1 kHz for wet kaolinite probed with Ag/AgCl electrodes). This observed polarization at 11.23 Hz may correspond to a spatial mechanism such as double-layer or membrane polarization. However, due to the dominance of electrode polarization at low frequencies, such features are obscured or hidden. Nevertheless, Figure 8a,b highlights spatial polarization features that remain observable above the *f*_EP_ threshold, where electrode polarization artifacts are extensively reduced.

We propose that the magnitude of the first derivative of in-phase conductivity (*∂σ*_eff_^′^/*∂*ω) or resistivity (*−∂ρ*_eff_^′^/*∂*ω) can be used to identify Pelton’s critical frequency, yielding *f*_c,P_ = 200 kHz (Figure 8c). In contrast, our circuit model yields a much lower critical frequency of *f*_c,P_ = 11.23 Hz. This discrepancy reflects different spatial polarization scales captured by each approach. Lower frequencies are associated with larger spatial scales (e.g., trapped free ions and counterions within pores), whereas higher frequencies correspond to smaller spatial features (e.g., counterions in diffuse layer).

Equations 24 and 25 are not equivalent. While they describe similar relaxation behavior in conductivity forms, the relaxation times in the resistivity-based Pelton’s model (τ_P_) and the Cole–Cole’s model (τ_CC_) differ but are related by^39,40^

$$\tau_{\mathrm{CC}}=\tau_{P}(1-m{)}^{1/\delta }\to f_{c,\mathrm{CC}}=\frac{f_{c,P}}{(1-m{)}^{1/\delta }}$$ 26

Assuming Pelton’s exponent ξ equals the Cole–Cole exponent δ, both *m* and δ collectively govern this relationship. With 0 < *m* < 1, it follows that τ_CC_ ≤ τ_P_ and thus, *f*_c,CC_ ≥ *f*_c,P_. Clay-rich media like lateritic soils often exhibit *m* ≈ 0.1–0.5 Volt/Volt. For *m* = 0.1 and δ = 1, τ_CC_ = 0.9τ_P_, so *f*_c,CC_ ≈ 1.1*f*_c,P_. For low-chargeability materials (e.g., deionized water, *m* = 0), τ_CC_ converges to τ_P_.

Since chargeability *m* is material-specific, a global critical frequency *f*_c_ must be defined. However, which peak frequency best represents Pelton’s *f*_c,P_ and the Cole–Cole model’s *f*_c,CC_? Prior studies often associate *f*_c,CC_ with the peak of admittance phase (−θ_*Z*_), quadrature resistivity (−ρ″), or quadrature conductivity (σ″).^67^ However, our wet kaolinite sample shows no clear peak in σ″ (Figure 9b). Instead, our Cole–Cole-derived *f*_c,CC_ provides the −ρ″ peak at 2 MHz (Figure 8c). Direct decomposition of the Cole–Cole model indicates global spatial polarization peaking at 422 kHz, with a dominant Maxwell–Wagner response at 1 MHz (Figure 8b). The *f*_c,CC_ values at 300 kHz, 1 MHz, and 25 MHz are possibly tied to double-layer, Maxwell–Wagner, and bound-water polarizations.

Figure 8d illustrates the estimated chargeability range *m* for δ = 0.75–1 (given η = 0.75 from Ag/AgCl measurements) across various *f*_c,CC_/*f*_c,P_ ratios. A previous discussion already reveals that Pelon’s exponent ξ equals the circuit model’s CPE exponent η. The ratio of 10 aligns well with chargeability values *m* = 0.83–0.86, consistent with the fundamental definition $m=\frac{\sigma_{\infty }-\sigma_{0}}{\sigma_{\infty }}$ (Figure 9a). Thus, the critical frequencies *f*_c,CC_ = 2 MHz, associated with the peak in −ρ^″^, and *f*_c,P_ = 200 kHz, determined from the maximum of *∂σ*_eff_^′^/*∂*ω or *−∂ρ*_eff_^′^/*∂*ω, can be considered characteristic of wet kaolinite.

The critical frequency inherently depends on the model employed; thus, variations across different models are to be expected. A future study should address what the different relaxation times represent at the molecular scale. Previous findings suggest that these variations may be associated with the polarizability of fluid molecules under confinement, which differs significantly from their behavior in the bulk phase.^68^ Influencing factors likely include the confinement size^6,29^ (e.g., pore size and the presence of clay forming ‘castle-like’ nano- to micro-structures), the type and content of clay (which contribute to surface charge effects),^6,69^ and the degree of saturation, which determines the proportion of bulk versus adsorbed water.^6,70^

## 5 Conclusions

This study has meticulously conducted analysis on wideband complex permittivity spectra spanning from 20 Hz to 1 GHz. The data from 20 Hz to 2 MHz is obtained using impedance spectroscopy, while data at higher frequencies (1 MHz–1 GHz) is measured using time domain reflectometry and the reflection decoupled ratio method. Analyses focus on fitting the data with Cole–Cole and circuit models to infer the inherent material polarizations and eliminate artifacts due to electrode polarization. Significant findings are as follows:

- The revisited theory unifies conductivity, permittivity, and impedance, deriving effective complex permittivity directly from impedance spectra without circuit assumptions. It elucidates the polarization–conduction interplay, extends permittivity models to heterogeneous media, and differentiates wave propagation from circuit regimes.
- The electrode polarization remains prevalent in impedance spectroscopy despite using Ag/AgCl probes, which are known to exhibit minimal electrode polarization.
- Nyquist plots (−*Z*″ vs *Z*′) are useful to detect following phenomena:
  1. Inherent material polarization typically forms a semicircular arc, which can generally be modeled with a parallel resistor–constant phase element (*R*∥CPE) circuit.
  2. Electrode polarization appears as a tail at higher resistance *Z*′ and can be modeled by placing an *R*∥CPE circuit in series with the semicircle associated with inherent material polarization.
  3. The limiting frequency where electrode polarization emerges, *f*_EP_, is seen as a local minimum of *Z*′ and shifts higher with more conductive materials or when using polarizable electrodes.
- In low-viscosity liquids that do not form glasses at room temperature, inherent material polarization is negligible below the orientational polarization frequency. In such cases, the observed real permittivity (κ′) values that greatly exceed the dielectric constant are attributed to electrode polarization. The permittivity spectra can be effectively fitted using both Cole–Cole and circuit models incorporating multiple relaxation processes.
- In contrast, wet soils present additional complexity. Although the Cole–Cole model can accommodate multiple relaxations to fit the permittivity spectra, there is currently no established benchmark to constrain the magnitude of spatial polarization effects (unknown Δκ_spatial_). Therefore, we employ circuit models that exclude electrode polarization to derive the true material impedance (*Z*_m_^*^) and extract the intrinsic permittivity spectrum.
- We have developed a method to determine the effective constant-phase element exponent $\left(\tilde{\eta }\right)$ by evaluating the slope of log permittivity over log frequency:
$$\tilde{\eta }=\pm \left(1+\frac{\partial \log\;\kappa^{\prime }}{\partial \log\;f}\right)$$
  This approach accounts for all possible combined exponents in the circuit models. The value of $\tilde{\eta }$ is frequency-dependent and influenced by the prevailing circuit components within a specific frequency regime.
- Chargeability of kaolinite derived from the fundamental definition, 0.83–0.86, agrees well with values derived from the ratio of critical frequencies *f*_c,CC_/*f*_c,P_ between the Cole–Cole and Pelton models. The Cole–Cole critical frequency *f*_c,CC_ can be identified from the peak of quadrature resistivity (−ρ″), while Pelton’s *f*_c, *P*_ corresponds to the peak of first derivative of effective in-phase resistivity $\left(-2\pi \frac{\partial \rho_{\mathrm{eff}}^{\prime }}{\partial \omega }\right)$ or effective real conductivity $\left(2\pi \frac{\partial \sigma_{\mathrm{eff}}^{\prime }}{\partial \omega }\right)$ .

## Supplementary Material





## Data Availability

Supporting Information is also available in the NYCU repository: https://dataverse.lib.nycu.edu.tw/dataverse/hakiki. We provide codes and data for figures. RDR codes for soil is available at DOI 10.57770/DL3WKS. RDR codes for isopropanol is available at DOI 10.57770/8EH963.

## Notes

Detailed formula derivations and extended analyses (PDF)
Data for main figures (XLSX)

## Notes

Farizal Hakiki has a legal name as Farizal Hakiki Soemarsono.
