# Supplementary material for: Broadband Complex Permittivity Spectra: Cole–Cole vs Circuit Models
Source: ACS Meas Sci Au. 2025 Sep 22;5(5):716–28. doi: 10.1021/acsmeasuresciau.5c00065 (PMC12532061; doi:10.1021/acsmeasuresciau.5c00065)
Supplement: Supplementary file 1 [file tg5c00065_si_001.pdf]

SUPPORTING INFORMATION

Broadband Complex Permittivity Spectra: Cole-Cole vs Circuit Models

Authors:
Farizal Hakiki [a,b,1,*], Chih-Ping Lin [a,b]

Affiliations:
[a] National Yang Ming Chiao Tung University, Disaster Prevention & Water Environment Research Center (DPWE), Hsinchu 300, Taiwan
[b] National Yang Ming Chiao Tung University, Civil Engineering Department, Hsinchu 300, Taiwan
[1]Fullname: Farizal Hakiki Soemarsono

*Corresponding author:
Farizal Hakiki Soemarsono (email: hakiki@nycu.edu.tw; alhakik@live.co.uk)

Published in *ACS Measurement Science Au*:
https://doi.org/10.1021/acsmeasuresciau.5c00065

Supporting Information is also available in the NYCU repository; included: data for main figures (in .xlsx), reflection decoupled ratio RDR codes, and high-resolution images:
https://dataverse.lib.nycu.edu.tw/dataverse/hakiki

RDR codes for soil: https://doi.org/10.57770/DL3WKS
RDR codes for isopropanol: https://doi.org/10.57770/8EH963

Table of Contents

1. **APPENDIX A: COLE-COLE MODEL DECOMPOSITION** 2
2. **APPENDIX B: CIRCUIT ELEMENT SENSITIVITY** 3
3. **APPENDIX C: COLE-COLE MODEL TO FIT ELECTRODE POLARIZATION** 8
4. **APPENDIX D: EFFECTIVE CPE EXPONENT η** 9
5. **APPENDIX E: PERMITTIVITY, CONDUCTIVITY, AND RESISTIVITY IN SPECTRAL FUNCTIONS** 15
6. **REFERENCES** 20

1. APPENDIX A: Cole-Cole Model Decomposition

Let's recall extended Cole-Cole model:

$$\varepsilon_{\text{eff}}^{*} = -j\frac{\sigma_{DC}}{\omega} + \varepsilon_{\infty} + \frac{\varepsilon_{s} - \varepsilon_{\infty}}{1 + (j\omega\tau)^{\delta}} \quad \text{[A1]}$$

also, let's consider Euler's identity: $j = e^{j\frac{\pi}{2}} = \cos\frac{\pi}{2} + j\sin\frac{\pi}{2}$ and its corresponding expanded forms:

$$j^{\delta} = e^{j\delta\frac{\pi}{2}} = \cos\delta\frac{\pi}{2} + j\sin\delta\frac{\pi}{2} \quad \text{[A2]}$$

$$(j\omega\tau)^{\delta} = (\omega\tau)^{\delta}\left(\cos\delta\frac{\pi}{2} + j\sin\delta\frac{\pi}{2}\right) \quad \text{[A3]}$$

$$1 + (j\omega\tau)^{\delta} = 1 + (\omega\tau)^{\delta}\cos\delta\frac{\pi}{2} + j(\omega\tau)^{\delta}\sin\delta\frac{\pi}{2} \quad \text{[A4]}$$

Then, we can simply express Eq. A4 as

$$1 + (j\omega\tau)^{\delta} = R + jT \quad \text{[A5]}$$

where $R = 1 + (\omega\tau)^{\delta}\cos\delta\frac{\pi}{2}$ and $T = (\omega\tau)^{\delta}\sin\delta\frac{\pi}{2}$. Therefore, we can rewrite the extended Cole-Cole model to become

$$\varepsilon_{\text{eff}}^{*} = -j\frac{\sigma_{DC}}{\omega} + \varepsilon_{\infty} + (\varepsilon_{s} - \varepsilon_{\infty})\frac{1}{R + jT} \quad \text{[A6a]}$$

$$\varepsilon_{\text{eff}}^{*} = -j\frac{\sigma_{DC}}{\omega} + \varepsilon_{\infty} + (\varepsilon_{s} - \varepsilon_{\infty})\frac{R - jT}{R^{2} + T^{2}} \quad \text{[A6b]}$$

$$\varepsilon_{\text{eff}}^{*} = \varepsilon_{\infty} + (\varepsilon_{s} - \varepsilon_{\infty})\ \frac{R}{R^{2} + T^{2}} - j\left[\frac{\sigma_{DC}}{\omega} + (\varepsilon_{s} - \varepsilon_{\infty})\ \frac{T}{R^{2} + T^{2}}\right] \quad \text{[A6c]}$$

Given that $\varepsilon_{\text{eff}}^{*} = \varepsilon' - j\varepsilon_{\text{eff}}''$, thus, the decomposed components (real: ε' and effective imaginary: $\varepsilon_{\text{eff}}''$) of the extended Cole-Cole model are

$$\varepsilon' = \varepsilon_{\infty} + (\varepsilon_{s} - \varepsilon_{\infty})\ \frac{R}{R^{2} + T^{2}} \quad \text{[A7]}$$

$$\varepsilon_{\text{eff}}'' = \frac{\sigma_{DC}}{\omega} + (\varepsilon_{s} - \varepsilon_{\infty})\ \frac{T}{R^{2} + T^{2}} \quad \text{[A8]}$$

2. APPENDIX B: Circuit Element Sensitivity

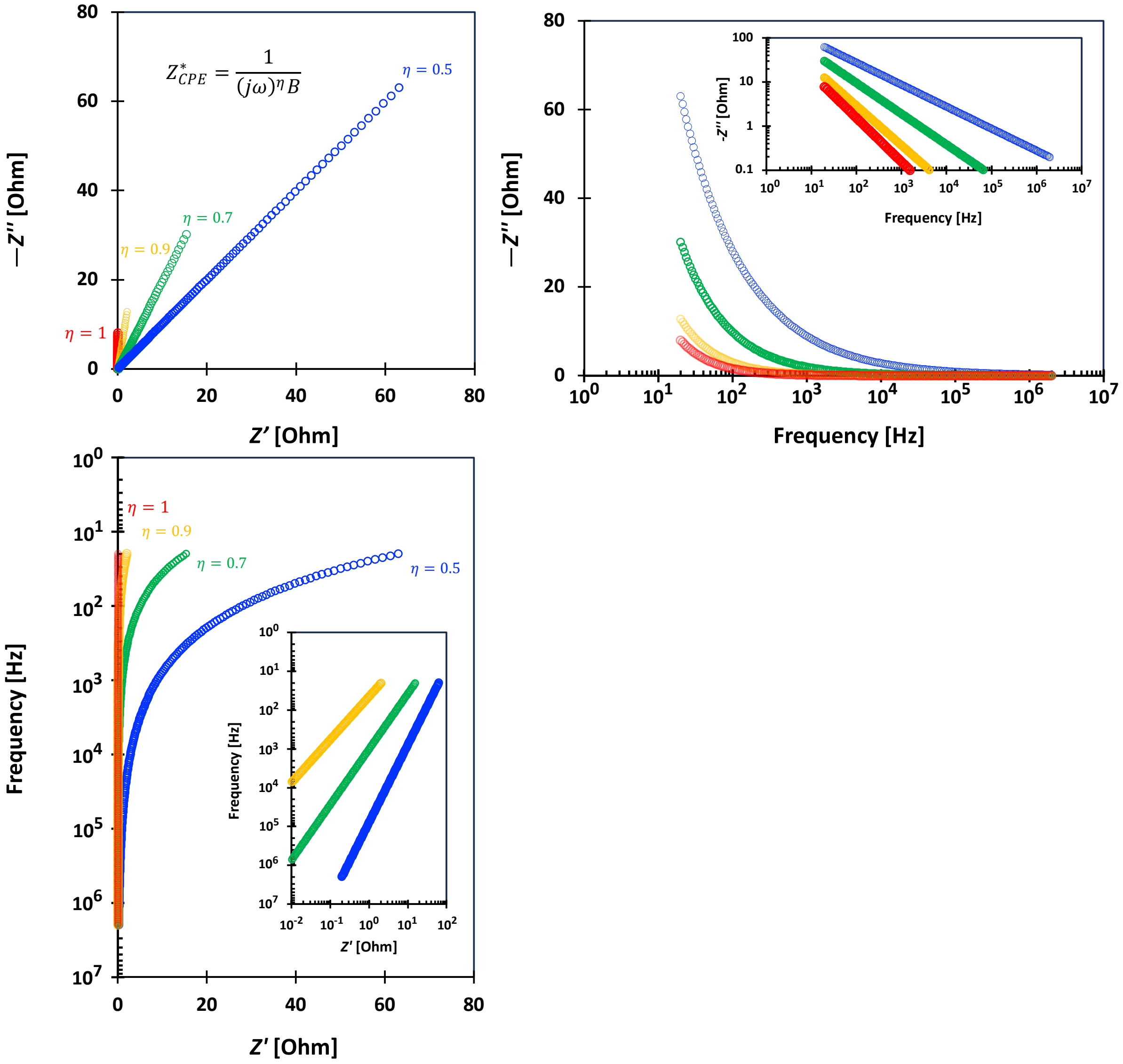

Figure S 1. Constant-Phase-Element CPE in Nyquist and spectral plots with varying CPE exponent η.

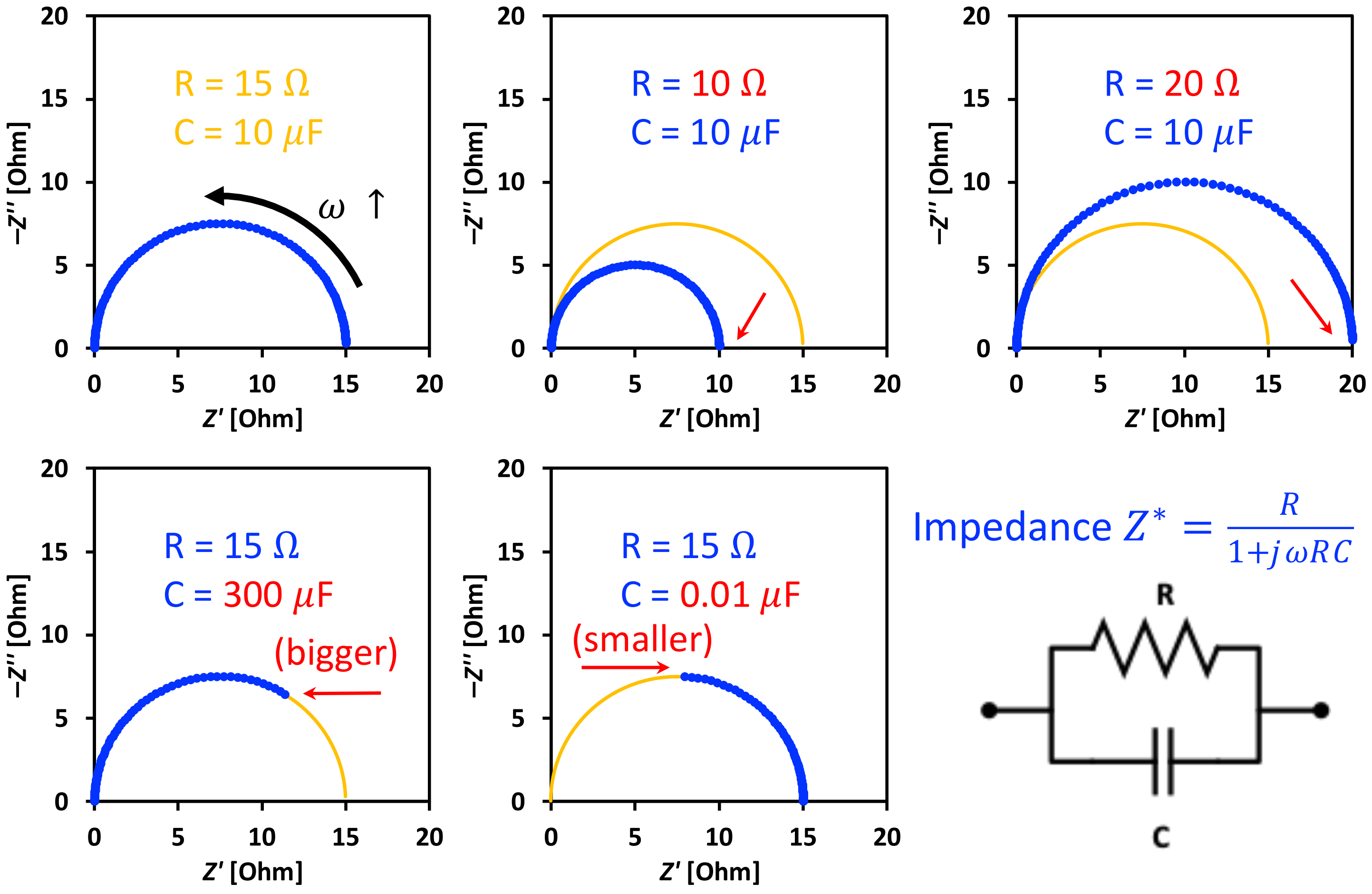

Figure S 2. Circuit model for R||C with variations in R and C. Frequency range: 20 Hz – 1 MHz, with the frequency sweep from the highest resistance Z′ to the lowest. All data are synthetic.

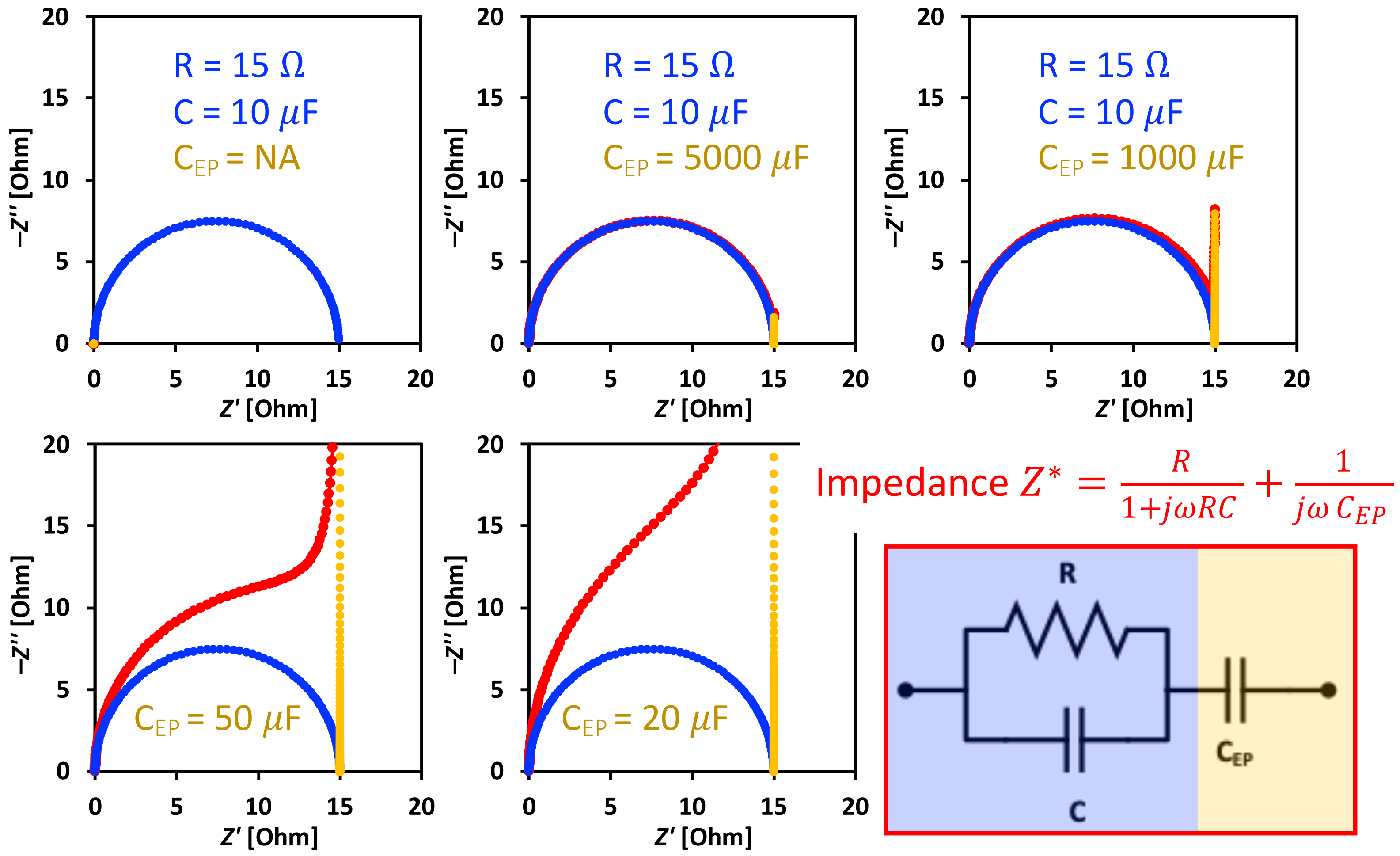

Figure S 3. Circuit model for R||C—C_{EP} with variations in C_{EP}. and is sweeping from the highest resistance Z' to the lowest. All data is synthetic. Subscript "EP" stands for the Electrode Polarization.

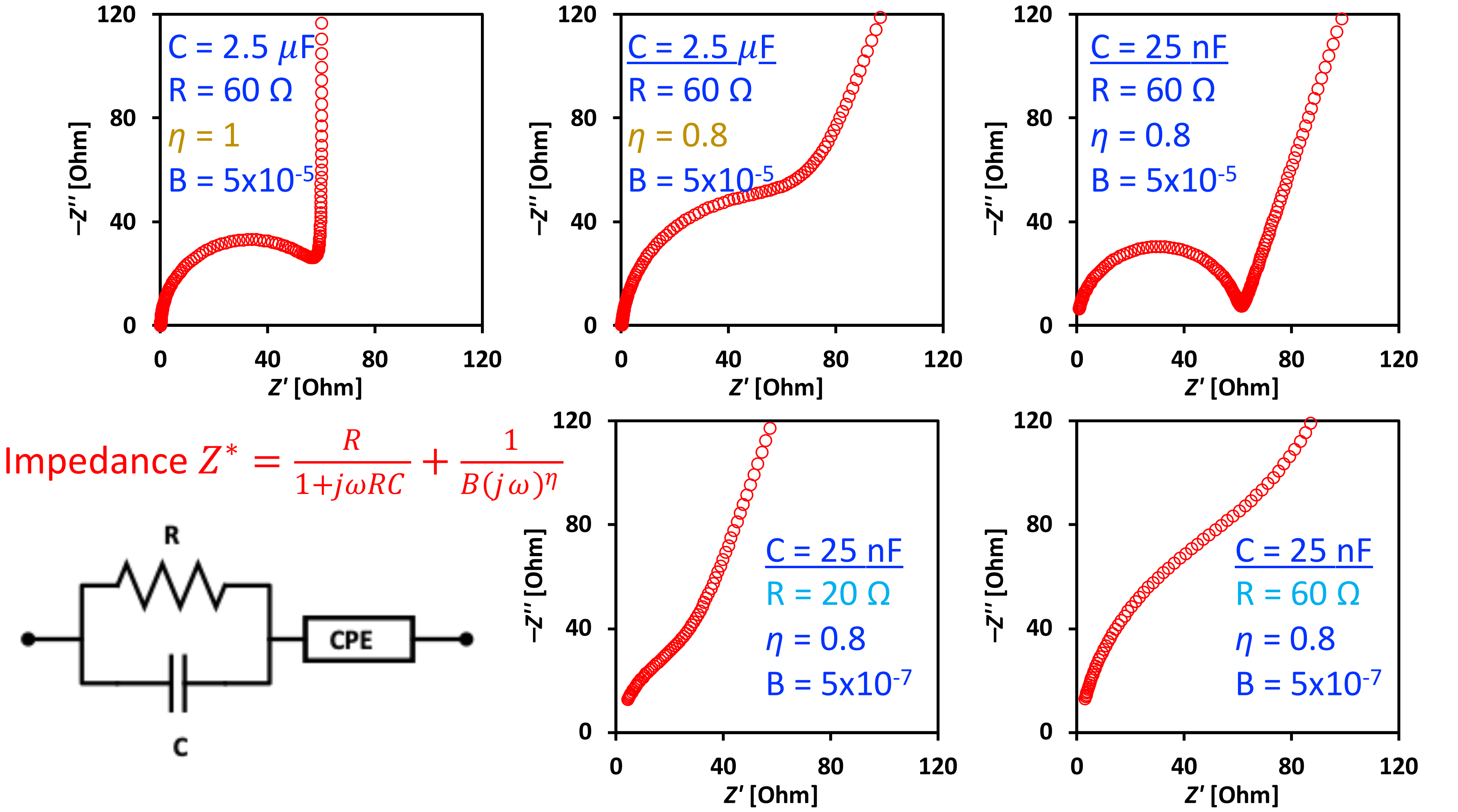

Figure S 4. Circuit model for R||C—CPE_{EP} with variations in R, C, η, and B_{EP}. and is sweeping from the highest resistance Z' to the lowest. All data is synthetic. Subscript "EP" stands for the Electrode Polarization.

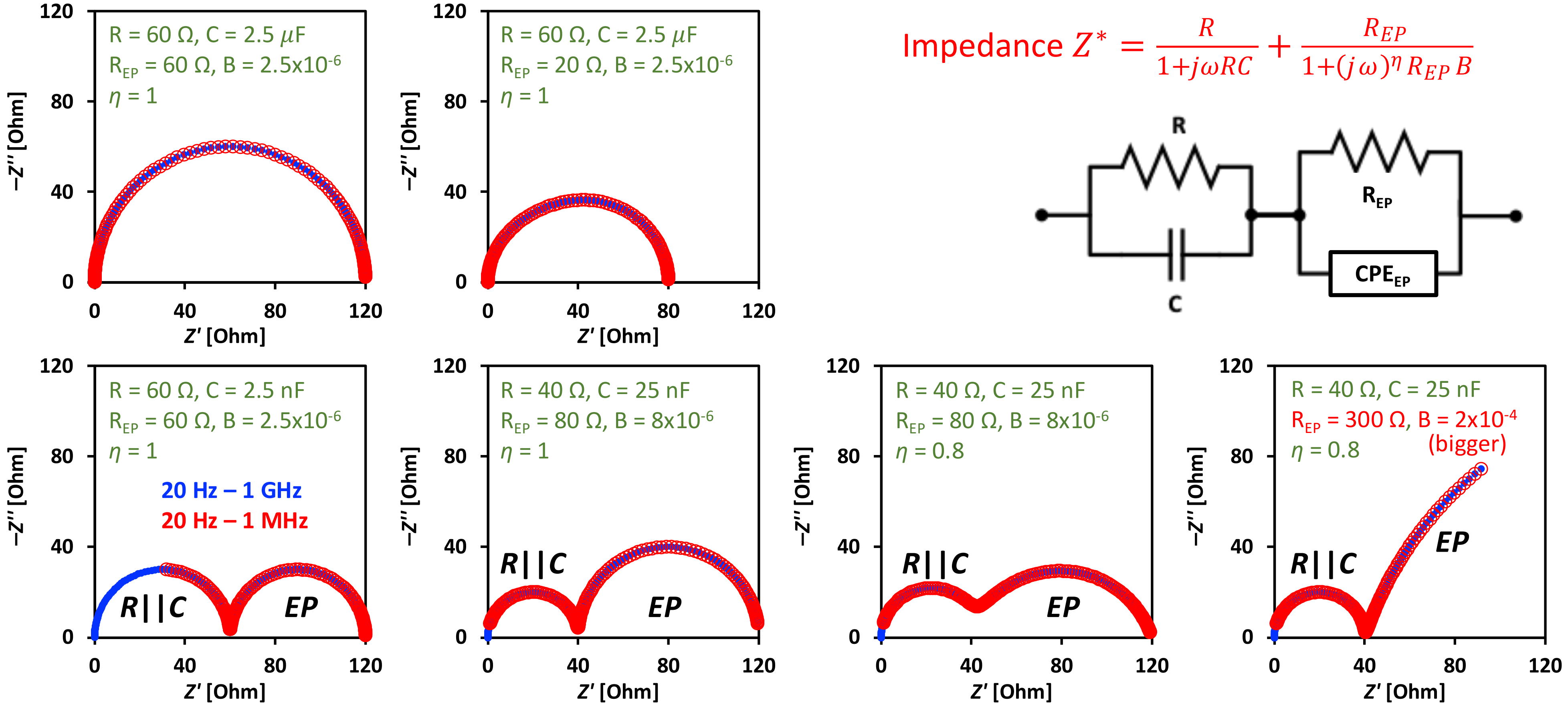

Figure S 5. Circuit model for R||C—R_{EP}||CPE_{EP} with variations in R, C, R_{EP}, B_{EP} and η. Red: Frequency range: 20 Hz – 1 MHz. Blue: Frequency range: 20 Hz – 1 GHz. All data is synthetic. Subscript "EP" stands for the Electrode Polarization.

3. APPENDIX C: Cole-Cole Model to Fit Electrode Polarization

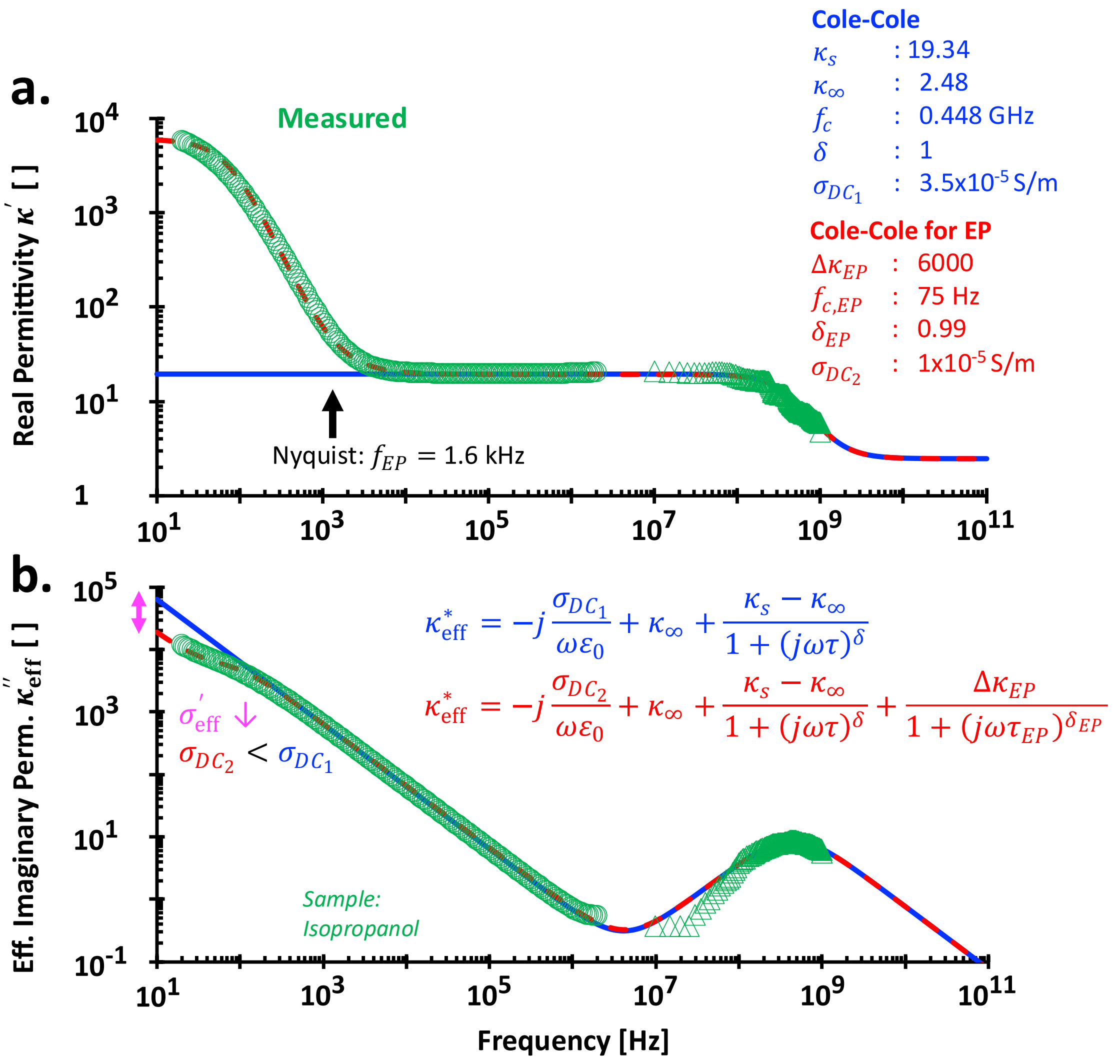

Figure S 6. Complex Permittivity Spectra vs. Extended Cole-Cole Models.

An extended Cole-Cole model that fits the complex permittivity spectra of isopropanol without the inclusion of electrode polarization (blue line):

$$\kappa^*_{\text{eff}} = -j\frac{\sigma_{DC_1}}{\omega\varepsilon_0} + \kappa_\infty + \frac{\kappa_s - \kappa_\infty}{1+(j\omega\tau)^\delta} \quad [C1]$$

where $\tau = 1/(2\pi f_c)$.

An extended Cole-Cole model that fits the complex permittivity spectra of isopropanol with the inclusion of electrode polarization (dashed red line):

$$\kappa_{\text{eff}}^* = -j\frac{\sigma_{DC_2}}{\omega\varepsilon_0} + \kappa_\infty + \frac{\kappa_s - \kappa_\infty}{1 + (j\omega\tau)^\delta} + \frac{\Delta\kappa_{EP}}{1 + (j\omega\tau_{EP})^{\delta_{EP}}} \quad \text{[C2]}$$

where $\tau_{EP} = 1/(2\pi f_{c,EP})$. Note that electrode polarization limiting frequency f_{EP} and the critical frequency of electrode polarization $f_{c,EP}$ are different.

4. APPENDIX D: Effective CPE Exponent $\tilde{\eta}$

Let us recall the basic equations involved in the derivation. The following equations were already derived and presented in the main manuscript. Effective complex permittivity $\varepsilon_{\text{eff}}^* = \kappa_{\text{eff}}^*\varepsilon_0 = \varepsilon' - j\varepsilon_{\text{eff}}'' = \varepsilon' - j\left(\varepsilon'' + \frac{\sigma_{DC}}{\omega}\right)$ could be computed from the admittance $Y^* = Y' + jY''$ such that

$$\begin{cases} \varepsilon' = \dfrac{Y''}{\beta\omega} \rightarrow \kappa' = \dfrac{Y''}{\beta\varepsilon_0\omega} \\ \varepsilon_{\text{eff}}'' = \dfrac{Y'}{\beta\omega} \rightarrow \kappa_{\text{eff}}'' = \dfrac{Y'}{\beta\varepsilon_0\omega} \end{cases} \quad \text{[D1]}$$

Complex impedance Z^* and admittance Y^* for a parallel form of resistor and CPE:

$$Z^* = \frac{1}{Y^*} = \frac{R}{1 + (j\omega)^\eta RB} = \frac{R}{1 + (j\omega\tau)^\eta}, \quad \tau = (RB)^{\frac{1}{\eta}} \quad \text{[D2]}$$

We use Euler's identity to expand $(j\omega\tau)^\eta$ term:

$$(j\omega\tau)^\eta = \omega^\eta\tau^\eta\left[\cos\left(\eta\frac{\pi}{2}\right) + j\sin\left(\eta\frac{\pi}{2}\right)\right] \quad \text{[D3]}$$

Therefore, the admittance of a parallel form of resistor and CPE:

$$Y^* = \frac{1 + (j\omega\tau)^\eta}{R} = \frac{1 + \omega^\eta\tau^\eta\cos\left(\eta\frac{\pi}{2}\right)}{R} + j\frac{\omega^\eta\tau^\eta\sin\left(\eta\frac{\pi}{2}\right)}{R} \quad \text{[D4]}$$

Subsequently, the relative complex permittivity become:

$$\begin{cases} \kappa' = \dfrac{Y''}{\beta\varepsilon_0\omega} = \dfrac{B\sin\left(\eta\dfrac{\pi}{2}\right)}{\beta\varepsilon_0\omega^{1-\eta}} \\ \kappa''_{\text{eff}} = \dfrac{Y'}{\beta\varepsilon_0\omega} = \dfrac{1}{\beta R\varepsilon_0\omega} + \dfrac{B\cos\left(\eta\dfrac{\pi}{2}\right)}{\beta\varepsilon_0\omega^{1-\eta}} \end{cases} \quad \text{[D5]}$$

The permittivity slope in a spectral form is proportional to the $(1-\eta)$ where η is the CPE exponent η such that

$$-\frac{\partial \log \kappa'}{\partial \log f} = 1 - \eta \quad \text{[D6]}$$

We have to call $\tilde{\eta}$ as an effective CPE exponent once we deal with multi-serial of $R||CPE$ models. The effective exponent $\tilde{\eta}$ values are frequency-dependent and varies based on the slope of the permittivity spectrum, such that $\tilde{\eta} = 1 + \frac{\partial \log \kappa'}{\partial \log f}$, and rely on which circuit components are the most influencing at certain frequency regime.

We can express impedance Z^* consists of a series of two $R||CPE$ circuits to be

$$Z^* = \frac{R_1}{1 + (j\omega\tau_1)^{\eta_1}} + \frac{R_2}{1 + (j\omega\tau_2)^{\eta_2}} \quad \text{[D7]}$$

Let $\alpha = (j\omega\tau_1)^{\eta_1}$ and $\beta = (j\omega\tau_2)^{\eta_2}$, such that the impedance and admittance are

$$Z^* = \frac{R_1}{1+\alpha} + \frac{R_2}{1+\beta} = \frac{R_1(1+\beta) + R_2(1+\alpha)}{1+\alpha+\beta+\alpha\beta} \quad \text{[D8]}$$

$$Y^* = \frac{1}{Z^*} = \frac{1+\alpha+\beta+\alpha\beta}{R_1(1+\beta)+R_2(1+\alpha)} = \frac{1+\alpha+\beta+\alpha\beta}{R_1+R_2+R_1\beta+R_2\alpha} \quad \text{[D9]}$$

We define the numerator $A = A_r + jA_i = 1 + \alpha + \beta + \alpha\beta$ and denominator $B = B_r + jB_i$ to redefine the admittance Y^* such that

$$Y^* = \frac{A_r + jA_i}{B_r + jB_i} = \frac{(A_r + jA_i)(B_r - jB_i)}{(B_r + jB_i)(B_r - jB_i)} = \frac{(A_rB_r + A_iB_i) + j(A_iB_r - A_rB_i)}{B_r^2 + B_i^2} \quad \text{[D10]}$$

Thus,

$$\begin{cases} \kappa' = \dfrac{Y''}{\beta\varepsilon_0\omega} = \dfrac{A_iB_r - A_rB_i}{\beta\varepsilon_0\omega}\dfrac{1}{B_r^2 + B_i^2} \\ \kappa''_{\text{eff}} = \dfrac{Y'}{\beta\varepsilon_0\omega} = \dfrac{A_rB_r + A_iB_i}{\beta\varepsilon_0\omega}\dfrac{1}{B_r^2 + B_i^2} \end{cases} \quad \text{[D11]}$$

To simplify the writing, we just write $(j\omega\tau)^\eta = \omega^\eta\tau^\eta(c + js)$ where $c = \cos\left(\eta\frac{\pi}{2}\right)$ and $s = \sin\left(\eta\frac{\pi}{2}\right)$. The expansion of numerator A is

$$A = 1 + \alpha + \beta + \alpha\beta = 1 + (j\omega\tau_1)^{\eta_1} + (j\omega\tau_2)^{\eta_2} + (j\omega\tau_1)^{\eta_1}(j\omega\tau_2)^{\eta_2} \rightarrow$$

$$A = 1 + \omega^{\eta_1}\tau_1^{\eta_1}(c_1 + js_1) + \omega^{\eta_2}\tau_2^{\eta_2}(c_2 + js_2) \quad \text{[D12]}$$

$$+ \omega^{\eta_1+\eta_2}\tau_1^{\eta_1}\tau_2^{\eta_2}(c_1 + js_1)(c_2 + js_2)$$

In the end, we can achieve

$$A = f(k_A, \omega^{\eta_1}, \omega^{\eta_2}, \omega^{\eta_1+\eta_2}) = \begin{cases} A_r = f\left(k_{A_r}, \omega^{\eta_1}, \omega^{\eta_2}, \omega^{\eta_1+\eta_2}\right) \\ A_i = f\left(k_{A_i}, \omega^{\eta_1}, \omega^{\eta_2}, \omega^{\eta_1+\eta_2}\right) \end{cases} \quad \text{[D13]}$$

where A is a summation function of constant k_A and these following frequency-dependent terms: ω^{η_1}, ω^{η_2}, and $\omega^{\eta_1+\eta_2}$. Let's also expand the denominator B become $B = R_1 + R_2 + R_1\beta + R_2\alpha = R_1 + R_2 + R_1(j\omega\tau_2)^{\eta_2} + R_2(j\omega\tau_1)^{\eta_1}$ and is equivalent to:

$$B = \left(R_1 + R_2 + R_1\omega^{\eta_2}\tau_2^{\eta_2}c_2 + R_2\omega^{\eta_1}\tau_1^{\eta_1}c_1\right) + j\left(R_1\omega^{\eta_2}\tau_2^{\eta_2}s_2 + R_2\omega^{\eta_1}\tau_1^{\eta_1}s_1\right) \quad \text{[D14]}$$

Finally, we achieve

$$B = f(k_B, \omega^{\eta_1}, \omega^{\eta_2}) = \begin{cases} B_r = f\left(k_{B_r}, \omega^{\eta_1}, \omega^{\eta_2}\right) \\ B_i = f\left(k_{B_i}, \omega^{\eta_1}, \omega^{\eta_2}\right) \end{cases} \quad \text{[D15]}$$

where B is a summation function of constant $k_B = k_{B_r} = R_1 + R_2$, $k_{B_i} = 0$, and these following frequency-dependent terms: ω^{η_1} and ω^{η_2}. Let's deduce term $B_r^2 + B_i^2 = (B_r + jB_i)(B_r - jB_i)$ that can be summarized as follow:

$$B_r^2 + B_i^2 = f_i\left(k_{|B|}, \omega^{\eta_1}, \omega^{\eta_2}, \omega^{2\eta_1}, \omega^{2\eta_2}, \omega^{\eta_1+\eta_2}\right) \quad \text{[D16]}$$

We also deduce the form of the term $(A_iB_r - A_rB_i)$ as follows

$$A_iB_r - A_rB_i = f_u(k'', \omega^{\eta_1}, \omega^{2\eta_1}, \omega^{\eta_2}, \omega^{2\eta_2}, \omega^{\eta_1+\eta_2}, \omega^{2\eta_1+\eta_2}, \omega^{\eta_1+2\eta_2}) \quad \text{[D17]}$$

Thus,

$$\kappa' = \frac{Y''}{\beta\varepsilon_0\omega} = \frac{1}{\beta\varepsilon_0\omega}\frac{f_u(k'', \omega^{\eta_1}, \omega^{2\eta_1}, \omega^{\eta_2}, \omega^{2\eta_2}, \omega^{\eta_1+\eta_2}, \omega^{2\eta_1+\eta_2}, \omega^{\eta_1+2\eta_2})}{f_l\left(k_{|B|}, \omega^{\eta_1}, \omega^{\eta_2}, \omega^{2\eta_1}, \omega^{2\eta_2}, \omega^{\eta_1+\eta_2}\right)} \quad \text{[D18]}$$

where the most influencing $\tilde{\eta}$ could be from upper f_u or lower f_l functions. If the $\tilde{\eta}$ from the denominator f_l's term prevails within a certain frequency, the permittivity spectral slope could be

$$-\frac{\partial \log \kappa'}{\partial \log f} = 1 + \tilde{\eta} \quad \text{[D19]}$$

and is equivalent to the negative form of Eq. D6, such that a general effective CPE exponent $\tilde{\eta}$ is redefined as:

$$\tilde{\eta} = \pm\left(1 + \frac{\partial \log \kappa'}{\partial \log f}\right) \quad \text{[D20]}$$

The values can be progressing in these possible options (not written in an order based on the sweeping frequency $\omega/2\pi$ nor the exponent magnitudes):

$$\tilde{\eta} = \pm\{\eta_1, \eta_2, 2\eta_1, 2\eta_2, \eta_1 + \eta_2, 2\eta_1 + \eta_2, \eta_1 + 2\eta_2\} \quad \text{[D21]}$$

The Table S 1 below helps identify possible values of $\tilde{\eta}$.

Table S 1. Possible $\tilde{\eta}$ values for two series of $R||CPE$. Each cell is summation between column (numerator A's exponents) and row (denominator B's exponents) components from a complex admittance $Y^* = A/B$.

A \ B	η_1	η_2	$\eta_1 + \eta_2$
η_1	$2\eta_1$	$\eta_1 + \eta_2$	$2\eta_1 + \eta_2$

η_2	$\eta_1 + \eta_2$	$2\eta_2$	$\eta_1 + 2\eta_2$

Possible η̃ values include their own components, e.g., exponents belonging to A (highlighted in yellow): η_1, η_2, and $\eta_1 + \eta_2$. Green cells are repeated symbols due to symmetrical effects onto the diagonal cells.

We can then express the impedance Z^* in a series of three $R||CPE$ circuits to be

$$Z^* = \frac{R_1}{1+\alpha} + \frac{R_2}{1+\beta} + \frac{R_3}{1+\gamma} \rightarrow$$

$$Z^* = \frac{B}{A} = \frac{R_1(1+\beta)(1+\gamma) + R_2(1+\alpha)(1+\gamma) + R_3(1+\alpha)(1+\beta)}{1+\alpha+\beta+\gamma+\alpha\beta+\beta\gamma+\alpha\gamma+\alpha\beta\gamma} \quad \text{[D22]}$$

where $\gamma = (j\omega\tau_3)^{\eta_3}$. The admittance $Y^* = \frac{1}{Z^*} = \frac{A}{B}$ can be summarized as follow

$$\begin{cases} A = f(k_A, \omega^{\eta_1}, \omega^{\eta_2}, \omega^{\eta_3}, \omega^{\eta_1+\eta_2}, \omega^{\eta_2+\eta_3}, \omega^{\eta_1+\eta_3}, \omega^{\eta_1+\eta_2+\eta_3}) \\ B = f(k_B, \omega^{\eta_1}, \omega^{\eta_2}, \omega^{\eta_3}, \omega^{\eta_1+\eta_2}, \omega^{\eta_2+\eta_3}, \omega^{\eta_1+\eta_3}) \end{cases} \quad \text{[D23]}$$

Eventually, the effective CPE exponent $\tilde{\eta}$ can be fluctuating in these values:

$$\tilde{\eta} = \pm f_1(\tilde{k}_1, \eta_1, \eta_2, \eta_3, \eta_1+\eta_2, \eta_2+\eta_3, \eta_1+\eta_3, \eta_1+\eta_2+\eta_3) \cdot f_2(\tilde{k}_2, \eta_1, \eta_2, \eta_3, \eta_1+\eta_2, \eta_2+\eta_3, \eta_1+\eta_3) \quad \text{[D24]}$$

Table S 2. Possible η̃ values for three series of R||CPE. Each cell is summation between column (numerator A's exponents) and row (denominator B's exponents) components from a complex admittance $Y^* = A/B$.

A \ B	η_1	η_2	η_3	$\eta_1+\eta_2$	$\eta_2+\eta_3$	$\eta_1+\eta_3$	$\eta_1+\eta_2+\eta_3$
η_1	$2\eta_1$	$\eta_1+\eta_2$	$\eta_1+\eta_3$	$2\eta_1+\eta_2$	$\eta_1+\eta_2+\eta_3$	$2\eta_1+\eta_3$	$2\eta_1+\eta_2+\eta_3$
η_2	$\eta_1+\eta_2$	$2\eta_2$	$\eta_2+\eta_3$	$\eta_1+2\eta_2$	$2\eta_2+\eta_3$	$\eta_1+\eta_2+\eta_3$	$\eta_1+2\eta_2+\eta_3$
η_3	$\eta_1+\eta_3$	$\eta_2+\eta_3$	$2\eta_3$	$\eta_1+\eta_2+\eta_3$	$\eta_2+2\eta_3$	$\eta_1+2\eta_3$	$\eta_1+\eta_2+2\eta_3$
$\eta_1+\eta_2$	$2\eta_1+\eta_{2,}$	$\eta_1+2\eta_2$	$\eta_1+\eta_2+\eta_3$	$2\eta_1+2\eta_2$	$\eta_1+2\eta_2+\eta_3$	$2\eta_1+\eta_2+\eta_3$	$2\eta_1+2\eta_2+\eta_3$
$\eta_2+\eta_3$	$\eta_1+\eta_2+\eta_3$	$2\eta_2+\eta_3$	$\eta_2+2\eta_3$	$\eta_1+2\eta_2+\eta_3$	$2\eta_2+2\eta_3$	$\eta_1+\eta_2+2\eta_3$	$\eta_1+2\eta_2+2\eta_3$
$\eta_1+\eta_3$	$2\eta_1+\eta_3$	$\eta_1+\eta_2+\eta_3$	$\eta_1+2\eta_3$	$2\eta_1+\eta_2+\eta_3$	$\eta_1+\eta_2+2\eta_3$	$2\eta_1+2\eta_3$	$2\eta_1+\eta_2+2\eta_3$

Possible η̃ values include their own components, e.g., exponents belonging to A (highlighted in yellow). Green cells are repeated symbols due to symmetrical effects onto the diagonal cells.

5. APPENDIX E: Permittivity, Conductivity, and Resistivity in Spectral Functions

Recalled Cole-Cole model in permittivity form [1]:

$$\varepsilon^* = \varepsilon_\infty + \frac{\varepsilon_s - \varepsilon_\infty}{1 + (j\omega\tau_{CC})^\delta} \quad \text{[E1]}$$

Due to the linear relationship between conductivity and permittivity: $\sigma^* = j\omega\varepsilon^*$, we can analogously substitute the ε_∞ with σ_∞ and ε_s with σ_0. Notably, the substitution is not due to a conversion because $\sigma_0 \neq \omega\varepsilon_s$ and $\sigma_\infty \neq \omega\varepsilon_\infty$. This is an arbitrary selection of boundary values. The $\sigma_0 = \sigma_{DC} = \omega\varepsilon''_{\mathrm{eff},\omega\approx 0}$ and $\varepsilon_s \neq \varepsilon''_{\mathrm{eff},\omega\approx 0}$. All of these ε_s, ε_∞, σ_0, and σ_∞ are obviously seen in each respective real (in-phase) component spectra. If we include the DC conductivity σ_{DC}, we address the conductivity-permittivity relationship to be $\sigma^*_{\mathrm{eff}} = j\omega\varepsilon^*_{\mathrm{eff}}$ where $\sigma^*_{\mathrm{eff}} = \sigma'_{\mathrm{eff}} + j\sigma''_{\mathrm{eff}}$, $\sigma'_{\mathrm{eff}} = \sigma_{DC} + \sigma'$, and $\sigma''_{\mathrm{eff}} = \sigma''$. Subscripts "0" and "∞" denote "DC" and "high-frequency" properties, respectively.

Eventually, we can alter Eq. E1: Cole-Cole model to be in a complex conductivity σ^*_{CC} as in an analogous form and also incorporating a chargeability m inclusion [2,3]:

$$\sigma^*_{CC} = \sigma_\infty + \frac{\sigma_0 - \sigma_\infty}{1 + (j\omega\tau)^\delta} = \sigma_\infty \left[1 + \frac{\left(\frac{\sigma_0 - \sigma_\infty}{\sigma_\infty}\right)}{1 + (j\omega\tau)^\delta}\right] = \sigma_\infty \left[1 - \frac{m}{1 + (j\omega\tau)^\delta}\right] \quad \text{[E2]}$$

where the dimensionless chargeability m is defined as [4]: $m = \frac{\sigma_\infty - \sigma_0}{\sigma_\infty} = 1 - \frac{\sigma_0}{\sigma_\infty} \rightarrow \sigma_\infty = \frac{\sigma_0}{1-m}$.

In a resistivity form, chargeability can be: $m = \frac{\sigma_\infty - \sigma_0}{\sigma_\infty} = \frac{\frac{1}{\rho_\infty} - \frac{1}{\rho_0}}{\frac{1}{\rho_\infty}} = 1 - \frac{\rho_\infty}{\rho_0} = \frac{\rho_0 - \rho_\infty}{\rho_0}$ and this implies that $\frac{\rho_0}{\rho_\infty} = \frac{1}{1-m}$ and $\rho_\infty = \rho_0(1 - m)$.

We can tweak Eq. E2 with $\sigma_\infty = \frac{\sigma_0}{1-m}$ and use a trick: $0 = -m + m$, so that

$$\sigma^*_{CC} = \frac{\sigma_0}{1 - m}\left[1 - m + m - \frac{m}{1 + (j\omega\tau_{CC})^\delta}\right] \quad \text{[E3]}$$

$$= \sigma_0 \left[1 + \frac{m}{1-m} - \frac{m}{1-m} \cdot \frac{1}{1 + (j\omega\tau_{CC})^\delta} \right]$$

$$= \sigma_0 \left[1 + \frac{m}{1-m} \left[1 - \frac{1}{1 + (j\omega\tau_{CC})^\delta} \right] \right]$$

Eq. E3 is preferable form over Eq. E2 due to the easiness to compute $\sigma_0 = \sigma_{DC}$. Subscript "CC" in Eq. E3 is to highlight that the complex conductivity is derived from Cole-Cole model.

Meanwhile, the complex resistivity ρ^* from Pelton's equation is expressed as [2,5]:

$$\rho_P^* = \rho_\infty + \frac{\rho_0 - \rho_\infty}{1 + (j\omega\tau_P)^\xi} = \rho_\infty \left[1 + \frac{\left(\frac{\rho_0}{\rho_\infty} - 1 \right)}{1 + (j\omega\tau_P)^\xi} \right]$$

$$= \rho_0 (1 - m) \left[1 + \frac{\frac{1}{1-m} - 1}{1 + (j\omega\tau_P)^\xi} \right]$$

$$= \rho_0 (1 - m) \left[1 + \frac{m}{1-m} \cdot \frac{1}{1 + (j\omega\tau_P)^\xi} \right] \quad \text{[E4]}$$

$$= \rho_0 \left[1 - m + \frac{m}{1 + (j\omega\tau_P)^\xi} \right]$$

$$= \rho_0 \left[1 - m \left[1 - \frac{1}{1 + (j\omega\tau_P)^\xi} \right] \right]$$

Equations E3 and E4 are definitely not in an analogous form. Therefore, we need to convert Eq. E4 into conductivity, play with algebraic tricks to rearrange and achieve analogous forms. We write relaxation time for Pelton's with τ_P and Cole-Cole's is τ_{CC}. To simplify the writing, let $u = (j\omega\tau_P)^\xi$. Therefore, Eq. E4 is equivalent with

$$\rho_P^* = \rho_0 \left[1 - m \left(1 - \frac{1}{1+u} \right) \right]$$

$$= \rho_0 \left(1 - m + \frac{m}{1+u} \right) \quad \text{[E5]}$$

$$= \rho_0 \left(\frac{1 + u - m - mu + m}{1 + u} \right)$$

$$= \rho_0 \left(\frac{1 + u - mu}{1 + u} \right)$$

$$= \rho_0 \left[\frac{1 + u(1 - m)}{1 + u} \right]$$

Now, let's invert the Pelton's equation in resistivity form ρ_P^* (Eq. E5) into conductivity form $\sigma_P^* = 1/\rho_P^*$ such that

$$\sigma_P^* = \sigma_0 \left[\frac{1 + u}{1 + u(1 - m)} \right] \quad \text{[E6]}$$

We can insert a zero ($0 = -mu + mu$) in the Eq. E6's numerator and a fractional trick later $\frac{1-m}{1-m}$ such that

$$\begin{aligned} \sigma_P^* &= \sigma_0 \left[\frac{1 + u - mu + mu}{1 + u(1 - m)} \right] \\ &= \sigma_0 \left[\frac{1 + u(1 - m) + mu}{1 + u(1 - m)} \right] \\ &= \sigma_0 \left[1 + \frac{mu}{1 + u(1 - m)} \right] \\ &= \sigma_0 \left[1 + \frac{m}{1 - m} \cdot \left[\frac{u(1 - m)}{1 + u(1 - m)} \right] \right] \end{aligned} \quad \text{[E7]}$$

Insert a zero ($0 = 1 - 1$) in the Eq. E7's second term numerator, so that

$$\begin{aligned} \sigma_P^* &= \sigma_0 \left[1 + \frac{m}{1 - m} \cdot \left[\frac{1 + u(1 - m) - 1}{1 + u(1 - m)} \right] \right] \\ &= \sigma_0 \left[1 + \frac{m}{1 - m} \cdot \left[1 - \frac{1}{1 + u(1 - m)} \right] \right] \end{aligned} \quad \text{[E8]}$$

Thus, the Pelton's equation in the conductivity form is expressed as

$$\sigma_P^* = \sigma_0 \left[1 + \frac{m}{1 - m} \cdot \left[1 - \frac{1}{1 + (j\omega\tau_P)^{\xi}(1 - m)} \right] \right] \quad \text{[E9]}$$

Let's now compare Cole-Cole (Eq. E3) and Pelton's model (Eq. E9) in Table S 3. Therefore, we can also obtain the complex permittivity $\varepsilon_{\mathrm{CC}}^{*}$ in the Cole-Cole and ε_{P}^{*} from Pelton's model.

If we assume both relaxation time exponents are the same $\delta = \xi$, denoted as μ, we can set a relation [2]:

$$(j\omega\tau_{CC})^{\mu} = (j\omega\tau_{P})^{\mu}(1-m)$$
$$\tau_{CC} = \tau_{P}(1-m)^{1/\mu} \quad \text{[E10]}$$

The relaxation time of Cole-Cole model would be equals to that of Pelton's model if the chargeability $m = 0$. Our tested wet kaolinite exhibits chargeability m ranges from 0.83 to 0.86 (Figure S 7). Conversion of net complex permittivity $\kappa_{\mathrm{eff}}^{*}$ spectra (by fitting) into complex conductivity $\sigma_{\mathrm{eff}}^{*}$ and resistivity ρ_{eff}^{*} are available in Figure S 7. We use an asymptotic extrapolated line to determine the σ_{∞} value.

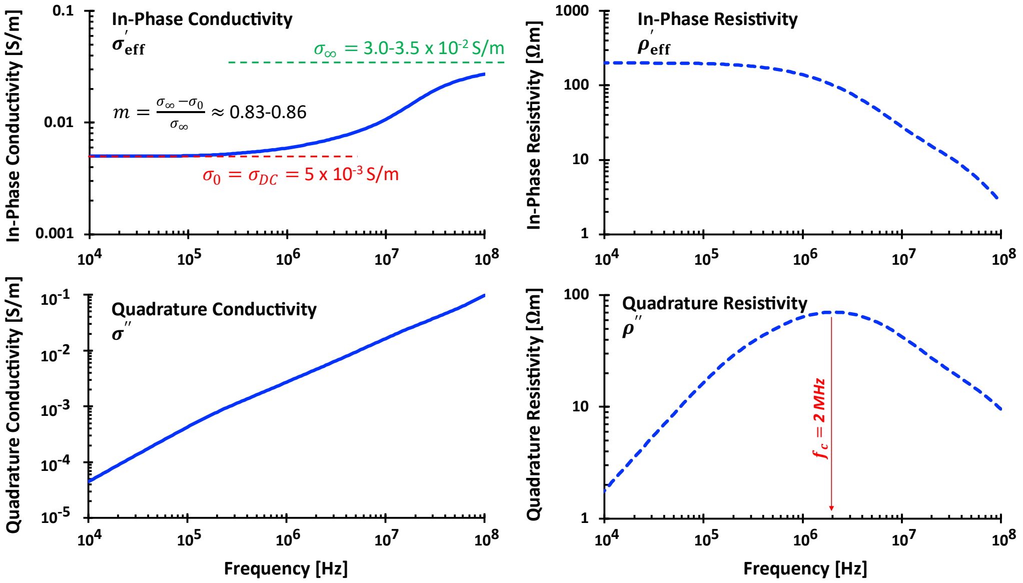

Figure S 7. Complex conductivity and resistivity spectra of wet kaolinite.

Table S 3. Pelton's and Cole-Cole models comparison.

Models in conductivity form:	Relaxation time terms	Chargeability
Pelton's $\sigma_P^* = \sigma_0 \left[1 + \frac{m}{1-m} \cdot \left[1 - \frac{1}{1+(j\omega\tau_P)^\xi(1-m)}\right]\right]$	$(j\omega\tau_P)^\xi(1-m)$	$m = \frac{\rho_0 - \rho_\infty}{\rho_0}$
Cole-Cole's $\sigma_{CC}^* = \sigma_0 \left[1 + \frac{m}{1-m}\left[1 - \frac{1}{1+\left(j\omega\tau_{\sigma,CC}\right)^\delta}\right]\right]$	$\left(j\omega\tau_{\sigma,CC}\right)^\delta$	$m = \frac{\sigma_\infty - \sigma_0}{\sigma_\infty}$
Models in permittivity form:	Relaxation time terms	Chargeability
Pelton's $\varepsilon_P^* = \frac{\sigma_P^*}{j\omega} = \frac{\sigma_0}{j\omega}\left[1 + \frac{m}{1-m} \cdot \left[1 - \frac{1}{1+(j\omega\tau_P)^\xi(1-m)}\right]\right]$	$(j\omega\tau_P)^\xi(1-m)$	$m = \frac{\sigma_\infty - \sigma_0}{\sigma_\infty}$
Cole-Cole's $\varepsilon_{CC}^* = \varepsilon_\infty + \frac{\varepsilon_s - \varepsilon_\infty}{1+\left(j\omega\tau_{\varepsilon,CC}\right)^\delta}$	$\left(j\omega\tau_{\varepsilon,CC}\right)^\delta$	N/A
Note: Cole-Cole relaxation times for conductivity and permittivity are not the same, $\tau_{\sigma,CC} \neq \tau_{\varepsilon,CC}$. They are only in analogous forms.		

6. REFERENCES

(1) Cole, K. S.; Cole, R. H. Dispersion and Absorption in Dielectrics I. Alternating Current Characteristics. *J Chem Phys* **1941**, *9* (4), 341–351. https://doi.org/10.1063/1.1750906.

(2) Tarasov, A.; Titov, K. On the Use of the Cole–Cole Equations in Spectral Induced Polarization. *Geophys J Int* **2013**, *195* (1), 352–356. https://doi.org/10.1093/gji/ggt251.

(3) Limbrock, J. K.; Kemna, A. Relationship between Cole–Cole Model Parameters in Permittivity and Conductivity Formulation. *Geophys J Int* **2024**, *239* (2), 964–970. https://doi.org/10.1093/gji/ggae300.

(4) Seigel, H. O. Mathematical Formulation and Type Curves for Induced Polarization. *Geophysics* **1959**, *24* (3), 547–565. https://doi.org/10.1190/1.1438625.

(5) Pelton, W. H.; Ward, S. H.; Hallof, P. G.; Sill, W. R.; Nelson, P. H. Mineral Discrimination and Removal of Inductive Coupling with Multifrequency IP. *GEOPHYSICS* **1978**, *43* (3), 588–609. https://doi.org/10.1190/1.1440839.
